# Repression of essential cell cycle genes increases cellular fitness

**DOI:** 10.1371/journal.pgen.1010349

**Published:** 2022-08-29

**Authors:** Michelle M. Conti, Julie M. Ghizzoni, Ana Gil-Bona, Wen Wang, Michael Costanzo, Rui Li, Mackenzie J. Flynn, Lihua Julie Zhu, Chad L. Myers, Charles Boone, Brenda J. Andrews, Jennifer A. Benanti

**Affiliations:** 1 Department of Molecular, Cell and Cancer Biology, University of Massachusetts Chan Medical School, Worcester, Massachusetts, United States of America; 2 Department of Computer Science and Engineering, University of Minnesota, Minneapolis, Minnesota, United States of America; 3 Donnelly Centre, University of Toronto, Toronto, Ontario, Canada; 4 Department of Molecular Genetics, University of Toronto, Toronto, Ontario, Canada; 5 Program in Bioinformatics and Integrative Biology, University of Massachusetts Chan Medical School, Worcester, Massachusetts, United States of America; 6 Program in Molecular Medicine, University of Massachusetts Chan Medical School, Worcester, Massachusetts, United States of America; University of California San Francisco, UNITED STATES

## Abstract

A network of transcription factors (TFs) coordinates transcription with cell cycle events in eukaryotes. Most TFs in the network are phosphorylated by cyclin-dependent kinase (CDK), which limits their activities during the cell cycle. Here, we investigate the physiological consequences of disrupting CDK regulation of the paralogous repressors Yhp1 and Yox1 in yeast. Blocking Yhp1/Yox1 phosphorylation increases their levels and decreases expression of essential cell cycle regulatory genes which, unexpectedly, increases cellular fitness in optimal growth conditions. Using synthetic genetic interaction screens, we find that Yhp1/Yox1 mutations improve the fitness of mutants with mitotic defects, including condensin mutants. Blocking Yhp1/Yox1 phosphorylation simultaneously accelerates the G1/S transition and delays mitotic exit, without decreasing proliferation rate. This mitotic delay partially reverses the chromosome segregation defect of condensin mutants, potentially explaining their increased fitness when combined with Yhp1/Yox1 phosphomutants. These findings reveal how altering expression of cell cycle genes leads to a redistribution of cell cycle timing and confers a fitness advantage to cells.

## Author summary

Proper regulation of the cell division cycle is essential to prevent uncontrolled proliferation that can lead to the development of cancer. Although numerous proteins have been characterized that control this process, mutations can sometimes have unexpected effects on cell proliferation. Here, we investigate mutations in two partially-redundant transcriptional repressors that control the cell cycle in yeast. Mutations that increase expression of these repressors result in decreased expression of essential cell cycle genes and, surprisingly, increase cellular fitness. We find that by regulating the expression of cell cycle genes these TFs control the amount of time cells spend in each cell cycle phase. Our results show that mutant cells increase the length of mitosis without decreasing proliferation rate, and as a result, mutant cells have more time to correct errors in chromosome segregation. Our findings demonstrate how mutations that impair progression of specific phases of the cell cycle can confer a fitness advantage to cells.

## Introduction

The cell division cycle is driven by a highly regulated pattern of periodic gene expression, which coordinates diverse cellular processes with cell division [1–3]. This gene expression pattern is established by a conserved network of cell cycle regulatory transcription factors (TFs), which are themselves regulated by phosphorylation and protein degradation to limit their activities to specific windows of the cell cycle [4–6]. Proper regulation of this network in human cells is crucial to protect the integrity of the genome and prevent uncontrolled cell division. Importantly, disruption of this regulatory network contributes to the development of cancer [7,8].

The functions of cell cycle regulatory TFs and the molecular consequences of their phosphorylation are well-defined in budding yeast [4]. Similar to regulation of cell cycle TFs in mammalian cells, phosphorylation by cyclin-dependent kinase (Cdk1) inactivates yeast transcriptional repressors and stimulates the functions of transcriptional activators to drive gene expression in specific cell cycle phases. At the G1/S transition, Cdk1 phosphorylation of the repressor Whi5 releases it from SBF-bound promoters and relocalizes it to the cytoplasm to allow transcription of genes required for S phase [9–11]. Subsequently, in S phase, Cdk1 phosphorylation activates the activator Hcm1, and promotes degradation of the repressors Yhp1 and Yox1 [12]. At the G2/M transition, Cdk1 phosphorylation activates the Fkh2/Ndd1 complex to promote expression of mitotic genes [13,14]. Interestingly, although mutation of Cdk1 phosphosites in most of these TFs disrupts the timing of their degradation and/or their transcriptional regulatory functions, phosphoregulation of many TFs is not essential for cell cycle progression, and mutations in many phosphosites only modestly affect gene expression. Moreover, some evidence suggests that cyclical genes exhibit periodic expression in absence of Cdk1 activity [15,16], although this result is controversial [17]. Therefore, the importance of TF phosphorylation for controlling cell cycle progression remains unclear.

One example where disrupting phosphorylation by Cdk1 has unexpected consequences is the regulation of the two homeodomain TFs Yhp1 and Yox1. These partially redundant repressors are expressed in S phase when they shut off transcription of genes required for S phase entry and prevent premature expression of mitotic genes [18]. Cells lacking both repressors exhibit elevated expression of target genes across the cell cycle, bud at a smaller cell size, and enter S-phase earlier than wild type cells [18]. Cdk1 phosphorylation of each TF promotes degradation by the ubiquitin proteasome system and mutant proteins that cannot be phosphorylated exhibit increased expression and activity: their expression is prolonged over the cell cycle, increased levels of each TF are recruited to target gene promoters, and target gene expression is hyper-repressed [12]. Since many Yhp1/Yox1 target genes are essential for the cell cycle, a logical prediction is that proliferation should be slowed or inhibited in these cells. Surprisingly, this is not observed and phosphomutant cells proliferate at a similar rate to wild type cells. In addition, blocking phosphorylation of Yhp1/Yox1 rescues fitness defects in cells that have inactivating mutations in the S-phase transcriptional activator Hcm1 [12]. Given these observations, the importance of Yhp1/Yox1 phosphoregulation is unknown.

Here, we use cells expressing unphosphorylatable alleles of Yhp1 and Yox1 to examine how reduced expression of essential cell cycle genes impacts cellular function. Systematic genetic interaction screens revealed that reduced expression of Yhp1/Yox1 target genes affects multiple cellular processes. Unphosphorylatable Yhp1/Yox1 mutants exhibit negative genetic interactions with factors required for transcription, consistent with the fact that transcription mutants further reduce expression of Yhp1/Yox1 target genes that are essential for the cell cycle. Surprisingly, blocking Yhp1/Yox1 phosphorylation increased the fitness of strains that exhibit mitotic defects. Most notably, positive genetic interactions were uncovered with the condensin complex, which is an essential regulator of chromosome segregation during mitosis. We find that phosphoregulation of Yhp1/Yox1 is required to establish the proper duration of cell cycle phases. Our data suggest that reducing expression of Yhp1/Yox1 target genes accelerates the G1/S transition and slows mitotic exit, enabling cells to better cope with chromosome segregation problems, such as those that occur in condensin mutants. These findings demonstrate that phosphoregulation of cell cycle TFs by Cdk1 helps to establish the length of cell cycle phases and, by extension, regulates cellular fitness.

## Results

### Genetic interaction screens identify consequences of Yhp1/Yox1 phosphorylation

**Fig 1:**
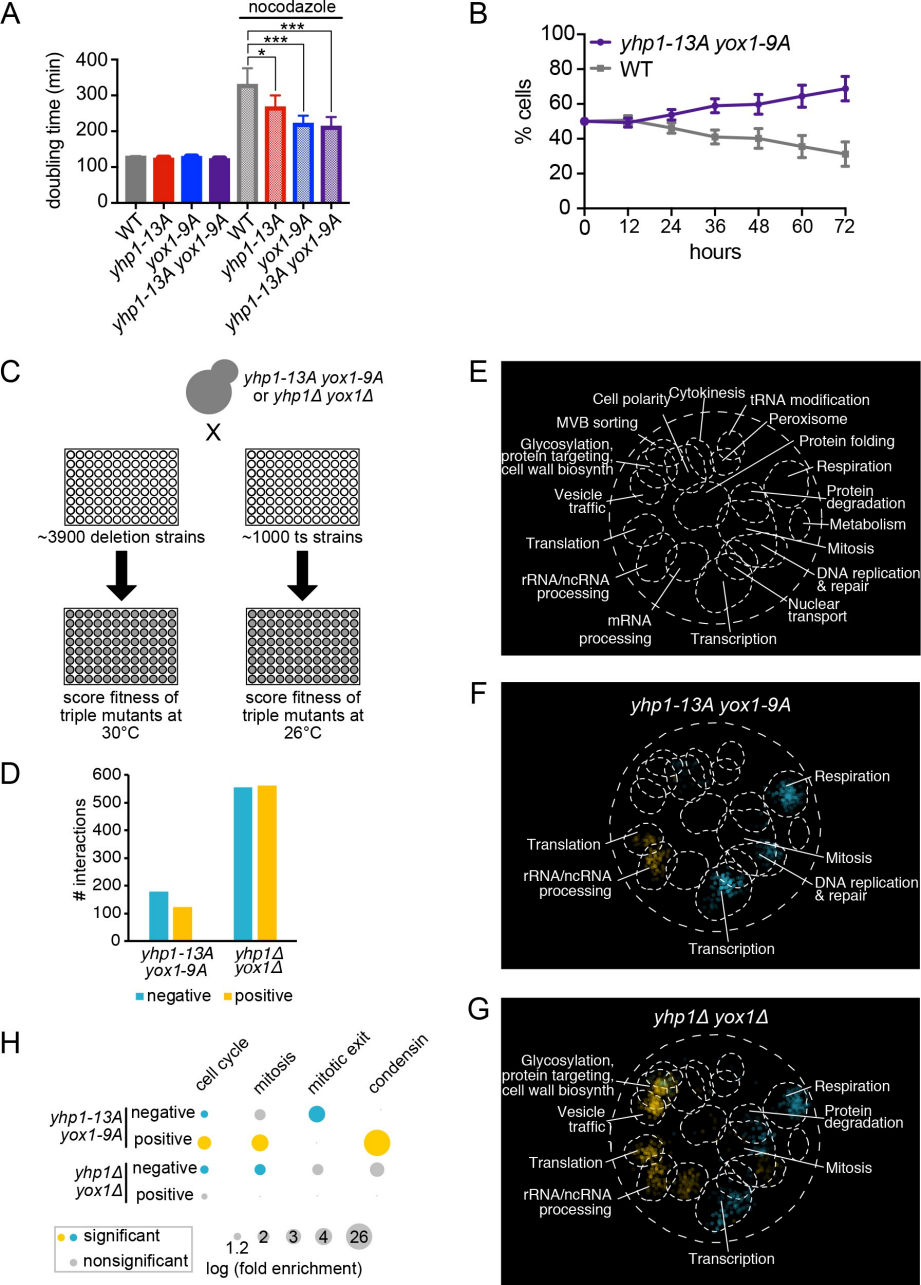
Identification of genetic interactions with *yox1 yhp1* mutant strains. **(A)** Doubling times of wild type (WT) or mutant strains with the indicated genotypes grown in rich media without (solid bars) or with (speckled bars) 5 μg/ml nocodazole at 30°C. Shown is an average of n = 6 experiments, error bars indicate standard deviations. Significance was tested using an unpaired t-test, *p < 0.05, ***p ≤ 0.0005. **(B)** WT and *yhp1-13A yox1-9A* strains were co-cultured, diluted every 12 hours to maintain logarithmic growth, and the percentage of each strain was quantified at the indicated time points. Shown is an average of n = 10 pairs of independently derived strains (each measured in duplicate or triplicate), error bars indicate standard deviations. See S1D Fig for plots of all individual experiments. Wild type—wild type control experiments are included in S1B and S1C Fig. **(C)** Outline of genetic interaction screens. Two screens were performed for each double mutant query strain (*yhp1-13A yox1-9A* and *yhp1Δ yox1Δ)*: one with non-essential deletion strains and one with temperature-sensitive (ts) alleles of essential genes. Growth of the resulting triple mutants was then inferred from colony size of cells growing on plates. **(D)** Number of negative (blue) and positive (yellow) interactions identified in *yhp1-13A yox1-9A* and *yhp1Δ yox1Δ* screens. Interacting genes identified in screens with non-essential and essential genes are combined. **(E)** Depiction of the previously described global genetic interaction network, visualized using SAFE analysis [19]. Regions enriched for specific biological processes are indicated. **(F-G)** SAFE analysis of interacting genes in *yhp1-13A yox1-9A* (F) and *yhp1Δ yox1Δ* (G) screens. Named processes are significantly enriched. Blue indicates negative interactions, yellow indicates positive interactions. **(H)** Enrichment of genes in specific cell cycle lists in the indicated groups of interacting genes. Node size represents log fold enrichment, colors represent significant interactions with p < 0.05. For parts (F-H) all analyses and statistics are included in S2 and S3 Datasets. Genes included in curated gene lists in part (H) are included in S4 Dataset.

Simultaneous mutation of Cdk1 phosphorylation sites in Yhp1 and Yox1 (*yhp1-13A* and *yox1-9A*, respectively) was previously found to have no detectable effect on the proliferation rate of cells growing on agar plates [12]. To examine the effect of these mutations on proliferation more precisely, the doubling times of strains expressing each mutant TF individually or together were first measured in liquid medium. Doubling times were nearly identical when cells were grown in rich medium (Fig 1A), similar to what was observed in plate-based assays. However, the *yhp1-13A yox1-9A* strain doubled significantly faster than wild type when cultures were treated with a sublethal dose of the microtubule poison nocodazole, consistent with previous findings [12].

To examine proliferation by a more sensitive approach, the double phosphomutant (*yhp1-13A yox1-9A*) was also assayed in a competitive growth assay with a wild type strain. In this assay, strains are co-cultured and diluted at regular intervals to maintain logarithmic growth. The relative frequencies of each strain in the population are followed over time using GFP markers: one strain expresses wild type GFP while the opposing strain expresses a non-fluorescent GFP mutant (Y66F). Because cells are co-cultured for many generations, this approach allows small differences in proliferation rate to be detected. Wild type strains expressing different GFP markers showed no difference in proliferation in this assay (S1A–S1C Fig), confirming that the GFP markers do not affect fitness. However, *yhp1-13A yox1-9A* strains exhibited a proliferative advantage compared to wild type when they were grown in co-culture (Fig 1B). Replicates of this experiment with 10 independently derived isolates of *yhp1-13A yox1-9A* supported this result (S1D Fig), indicating that the proliferative advantage was not the result of an unwanted mutation in a single strain. These results suggest that blocking phosphorylation of Yhp1 and Yox1 confers a fitness advantage to cells when they are growing in an optimal environment.

To understand how blocking phosphorylation of Yhp1/Yox1 can confer a fitness advantage, synthetic genetic array (SGA) analysis was performed to identify cellular processes that are positively or negatively impacted in phosphomutant cells [20,21]. The *yhp1-13A yox1-9A* query strain was crossed to ~3900 non-essential gene deletion strains and ~1000 strains carrying temperature-sensitive alleles of essential genes, representing ~600 genes [19,22] (Fig 1C). Colony size was used to infer fitness of the resulting triple mutant strains, with temperature-sensitive strains being screened at a semipermissive temperature where cells were viable but proliferated at a reduced rate. Genetic interactions were scored as negative if the resulting triple mutant strains had a fitness value that was less than the product of the combined defects of *yhp1-13A yox1-9A* and each individual deletion (or mutation). Positive genetic interactions were defined as triple mutants whose fitness was better than expected [19,23]. Altogether, 178 negative and 122 positive genetic interactions were identified with *yhp1-13A yox1-9A* (Fig 1D and S1 Dataset). To identify the processes that are impacted in *yhp1-13A yox1-9A* cells, several enrichment strategies were employed. Previously, data from genome-wide SGA screens were used to cluster genes with similar genetic interaction profiles [19]. Spatial analysis of functional enrichment (SAFE) analysis was then employed to functionally annotate gene clusters, generating a genetic map of the cell (Fig 1E). We similarly used SAFE analysis to identify enriched processes among the negative and positive interacting genes identified in our screens (see Methods). Negative genetic interactions were enriched for genes involved in transcription, respiration, mitosis, and DNA replication and repair, whereas positive interactions were enriched for genes regulating translation and rRNA/ncRNA processing (Fig 1F and S2 Dataset). Similar enrichments were identified using Gene Ontology (GO)-term enrichment analysis (S2 Dataset).

For comparison, complementary SGA screens were carried out with a double mutant query strain carrying deletions in both *YHP1* and *YOX1*. Deletion of both TFs increases expression of target genes [18], which is the opposite of what occurs in the *yhp1-13A yox1-9A* mutant. In comparison to the phosphomutant screens, the double deletion strain (*yhp1Δ yox1Δ*) interacted with approximately four times as many genes, with a total of 555 negative and 562 positive interactions (Fig 1D and S1 Dataset). Surprisingly, although the phosphomutant and deletion strains have opposite effects on gene expression, SAFE and GO term analyses revealed that many categories of genes that were enriched in their genetic interaction profiles overlapped (Fig 1F and 1G and S3 Dataset). These included negative interactions with transcription, respiration, and mitosis, as well as positive interactions with translation and rRNA/ncRNA processing. The *yhp1Δ yox1Δ* strain also interacted with additional categories of genes not observed in the phosphomutant. These categories included negative interactions with protein degradation genes, and positive interactions with genes involved in vesicle trafficking, and glycosylation, protein targeting and cell wall synthesis.

Although many categories of genes showed similar interactions with both deletion and phosphomutant strains, a closer examination of the data revealed that some genes known to be involved in cell cycle regulation displayed genetic interactions in opposite directions. Because cell cycle related categories (mitosis and DNA replication) that are identified by SAFE and enriched GO terms are very broadly defined, we also examined enrichment of interacting genes among curated lists of cell cycle, mitotic, and mitotic exit genes (see S4 Dataset for description). Surprisingly, although cell cycle genes were enriched among both positive and negative interactions in the *yhp1-13A yox1-9A* screens, the subset of these genes with mitosis-specific functions were significantly enriched specifically among the positive interactions. Conversely, mitotic genes were enriched specifically among negative interactions in *yhp1Δ yox1Δ* screens (Fig 1H). In addition to mitotic genes, we found that *yhp1-13A yox1-9A* negative interactions were also enriched for genes that regulate exit from mitosis. Since the phosphomutant strain exhibits a proliferative advantage compared to wild type (Fig 1B), we hypothesized that the observed genetic interactions between *yhp1-13A yox1-9A* and mitotic genes may be related to this increase in fitness.

### Identification of genes misregulated in phosphomutant cells

To better understand the identified genetic interactions, we examined genes that are misregulated in *yhp1-13A yox1-9A* cells. A set of Yhp1/Yox1 target genes was previously identified by profiling gene expression across the cell cycle in *yhp1Δ yox1Δ* cells [18], and several of these target genes are hyper-repressed in the *yhp1-13A yox1-9A* strain [12]. However, it was not known whether levels of all Yhp1/Yox1 targets are reduced in the phosphomutant strains, nor was it known whether Yhp1 and Yox1 phosphomutant proteins gained additional targets not regulated by their wild type counterparts. To address these questions, we performed RNA-seq on the *yhp1-13A yox1-9A* and *yhp1Δ yox1Δ* strains and identified changes in gene expression in each relative to wild type. Notably, relatively few genes significantly changed in expression in either *yhp1-13A yox1-9A* or *yhp1Δ yox1Δ* cells (S5 Dataset). Expression of target genes is expected to be reduced in *yhp1-13A yox1-9A* and increased in *yhp1Δ yox1Δ*. Only 16 genes fit this pattern and most of these genes changed in expression less than two-fold (S2A and S2B Fig). We reasoned that many target genes may not be identified as differentially expressed in this experiment if their oscillatory expression pattern, but not average expression, was affected. In addition, since these repressors have cell cycle stage-specific effects on target gene expression [12,18], it may be difficult to detect differential expression in asynchronous cultures.

**Fig 2:**
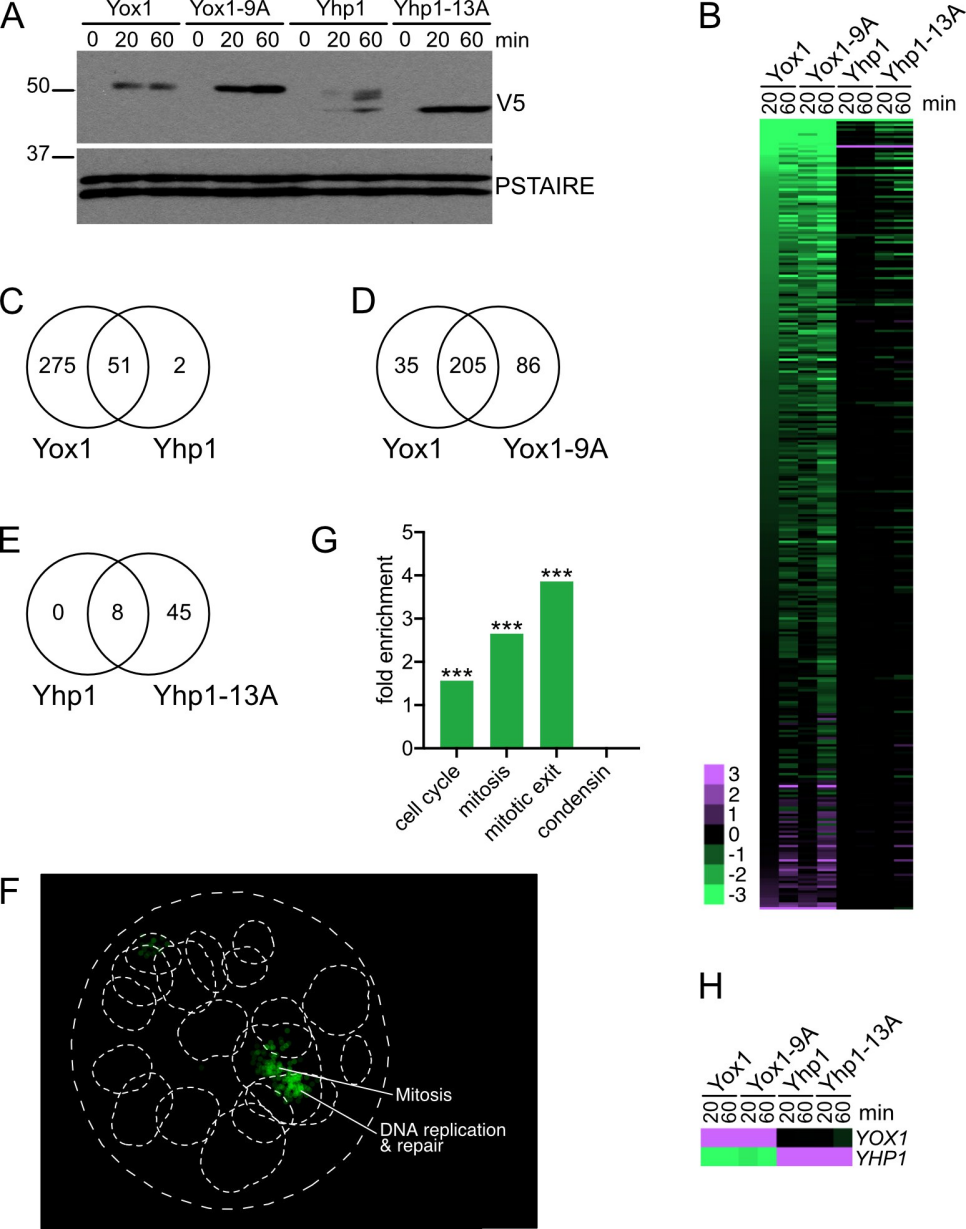
Identification of Yhp1/Yox1-regulated genes. Strains with the *Z3EV* promoter integrated upstream of *YHP1*, *yhp1-13A*, *YOX1*, or *yox1-9A* were treated with estradiol to induce TF overexpression for 20 or 60 minutes. **(A)** Western blot showing expression of the indicated proteins following 20 or 60 minutes of estradiol induction. All proteins were detected with antibodies that recognize a C-terminal 3V5 tag on Yhp1 or Yox1. PSTAIRE is shown as a loading control. **(B)** Heat map of all genes significantly changed in expression (at least 1.5-fold) after 20- or 60-minute induction of any of the four tested TF proteins. Genes are sorted by the background subtracted log fold change value following Yox1 induction after 20 minutes. List of genes and primary data is included in S6 Dataset. **(C)** Overlap of all genes that were significantly changed in any Yox1/Yox1-9A sample and all genes significantly changed in any Yhp1/Yhp1-13A sample. Overlap was determined to be significant using a hypergeometric test, p = 5.9e^-68^. **(D)** Overlap of all genes significantly changed in Yox1 at 20 or 60 minutes and all genes significantly changed in Yox1-9A at 20 or 60 minutes. Overlap was determined to be significant using a hypergeometric test, p = 6.4e^-270^. **(E)** Overlap of all genes significantly changed in Yhp1 at 20 or 60 minutes and all genes significantly changed in Yhp1-13A at 20 or 60 minutes. Overlap was determined to be significant using a hypergeometric test, p = 5.2e^-18^. **(F)** SAFE analysis of all genes shown in (B). Enriched processes are indicated. **(G)** Fold enrichment of genes from the indicated groups of cell cycle genes in the list of Yhp1/Yox1-regulated genes from (B), ***p < 0.0005. For parts (F) and (G) all analyses and statistics are included in S7 Dataset. **(H)** Zoom in on regulation of *YOX1* and *YHP1* expression from part (B), highlighting that Yox1 represses *YHP1* expression. Scale bar matches (B).

To overcome these issues and identify a list of likely target genes, we acutely overexpressed each TF and their phosphomutant counterparts from a synthetic, estradiol-inducible promoter [24]. In these strains, treatment with estradiol induced detectable expression of Yox1 and Yhp1 proteins within 20 minutes and maximal expression was achieved by 60 minutes (Fig 2A). As expected, phosphomutant proteins were expressed at higher levels than their wild type counterparts. These short induction times had little to no effect on the cell cycle distribution of the populations (S3 Fig). Notably, within 20 minutes several hundred genes were downregulated in one or more strains and very few genes were upregulated, consistent with the possibility that most downregulated genes are direct targets of the repressors (Fig 2B and S6 Dataset). All 28 genes that were initially identified as Yhp1/Yox1 targets through analysis of a *yhp1Δ yox1Δ* strain were included in this list [18], as well as 14 of the 16 genes that were identified in the comparison of *yhp1-13A yox1-9A* and *yhp1Δ yox1Δ* strains (S2 Fig). Interestingly, Yox1 repressed many more genes than Yhp1, even though the TFs were expressed at similar levels by the 60-minute time point. Moreover, almost all the Yhp1-regulated genes were a subset of the Yox1-regulated genes (Fig 2C), suggesting that the TFs repress many of the same genes, but that Yhp1 is a less potent repressor. Importantly, most genes repressed by Yox1 or Yhp1 were also repressed by their respective phosphomutants (Fig 2D and 2E), confirming that the mutations do not affect target gene specificity. Instead, the phosphosite mutations likely exert their effects by increasing Yhp1/Yox1 levels, leading to greater repression of their target genes.

We considered all genes that changed significantly (at least 1.5-fold) following acute Yhp1/Yox1 overexpression to be Yhp1/Yox1 target genes and performed enrichment analysis to identify cellular processes that are likely impacted in the phosphomutant strain. SAFE and GO term enrichment analyses confirmed that the primary functions of these TFs are to repress expression of genes involved in mitosis and DNA replication and repair (Fig 2F and S7 Dataset). In addition, genes in curated lists of cell cycle, mitotic, and mitotic exit genes were all significantly enriched among Yhp1/Yox1 targets (Fig 2G). A motif scan of the promoters of this set of genes also revealed a significant enrichment of Yox1, Fkh2, and Mcm1 motifs, supporting the possibility that many of these genes are cell cycle regulated and directly regulated by Yhp1/Yox1 (S8 Dataset). Finally, a regulatory relationship between Yox1 and Yhp1 was evident. Overexpression of Yox1 strongly repressed expression of Yhp1, although the converse did not occur (Fig 2H). This result suggests that the activities of Yox1 and Yhp1 are coordinated during the cell cycle.

### Negative interactions between *yhp1-13A yox1-9A* and conditional alleles of target genes

**Fig 3:**
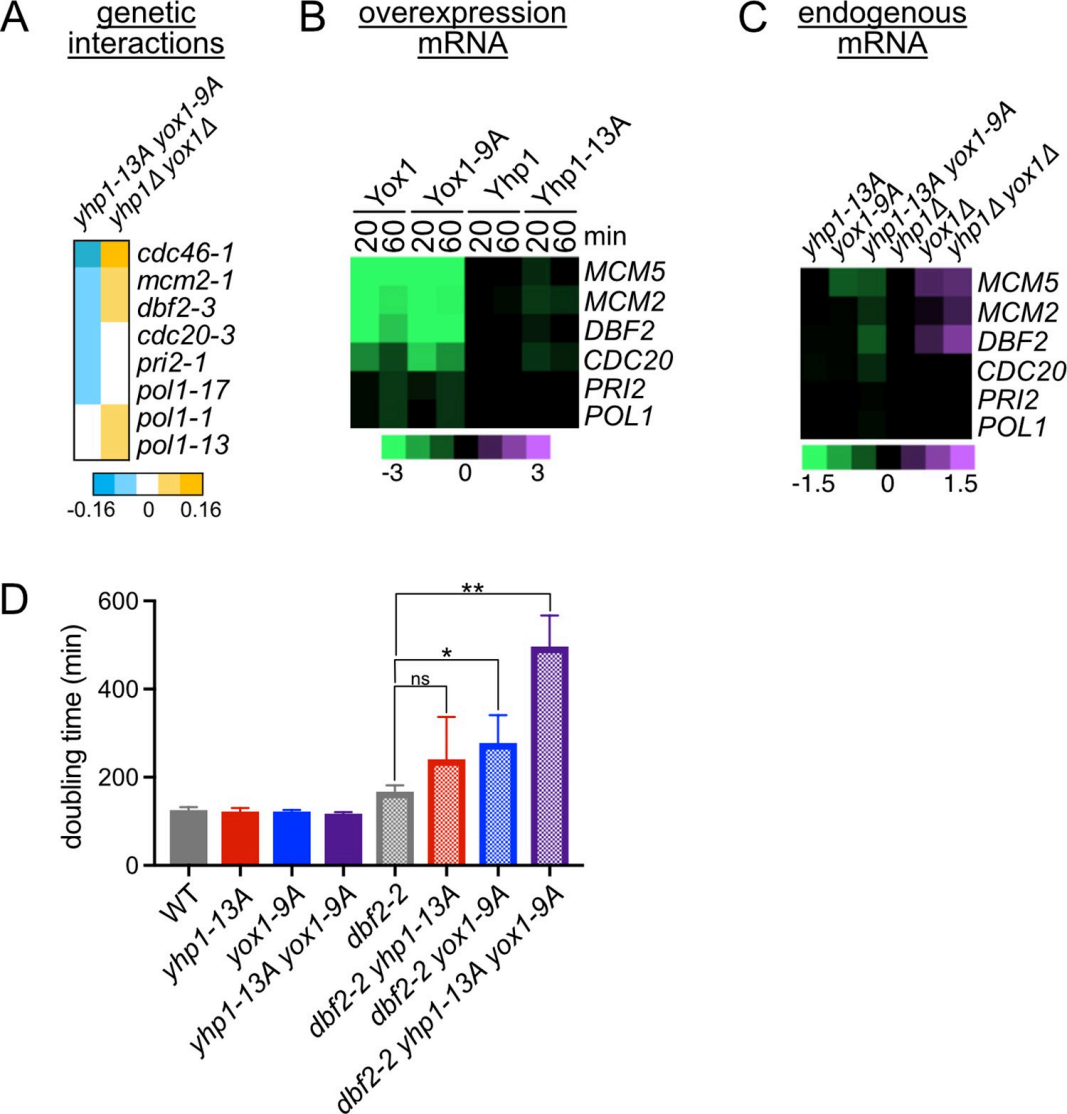
Negative interactions between *yhp1-13A yox1-9A* and conditional alleles of target genes. **(A)** Genetic interactions between double mutant query strains and conditional alleles of Yhp1/Yox1 target genes, from SGA screens described in Fig 1. **(B)** Gene expression of the indicated Yhp1/Yox1 target genes following acute overexpression of the indicated proteins, from RNAseq experiments in Fig 2. Negative genetic interactions were identified between ts alleles of these genes and *yhp1-13A yox1-9A*. **(C)** Expression of genes from (A) in strains expressing the indicated Yhp1/Yox1 mutants from their endogenous promoters, or deleted for the indicated TFs, from RNAseq experiments in S2 Fig. Note the difference in scale between (B) and (C). **(D)** Doubling times of the indicated strains growing at 30°C in rich media. Shown is an average of n = 3–5 replicates, error bars indicate standard deviations. Significance was tested using an unpaired t-test, *p < 0.05, **p < 0.005, ns = nonsignificant.

Since Yhp1/Yox1 repress many essential cell cycle regulatory genes, we expected to identify negative genetic interactions between *yhp1-13A yox1-9A* and hypomorphic alleles of those essential target genes (temperature sensitive alleles at semipermissive temperatures) in our SGA screen. Conversely, since deletion of *YHP1* and *YOX1* should increase expression of these genes, we expected to identify positive interactions between *yhp1Δ yox1Δ* and the same cell cycle regulatory genes. Consistent with these predictions, we identified negative interactions between *yhp1-13A yox1-9A* and temperature sensitive alleles of six genes that were found to be repressed by Yhp1/Yox1 (Fig 3A–3C). These genes include the replication proteins *MCM5/CDC46*, *MCM2*, *PRI2* and *POL1*, and the mitotic regulators *DBF2* and *CDC20*. We confirmed the negative interaction between *yhp1-13A yox1-9A* and *dbf2-2* (an alternate allele of *DBF2* that was not identified in the genetic interaction screen) and examined the relative contributions of the individual *yox1* and *yhp1* alleles to the phenotype by measuring doubling times. Notably, the individual *yhp1-13A* and *yox1-9A* alleles lengthened the doubling time of *dbf2-2* and the combination of alleles showed a stronger effect (Fig 3D). This is consistent with the observation that the double phosphomutant strain displayed a greater downregulation of *DBF2* gene expression compared to each single mutant (Fig 3C). Importantly, *yhp1Δ yox1Δ* displayed positive interactions with four of these genes (*MCM5/CDC46*, *MCM2*, *POL1* and *DBF2*), in line with our predictions (Fig 3A).

Interestingly, a second group of genes that displayed negative interactions with *yhp1-13A yox1-9A* were genes involved in transcription, including several subunits and/or regulators of RNA Polymerase II (Fig 1F and S1 Dataset). We validated the negative interaction with the mediator subunit *SRB2* and found significantly reduced expression of Yhp1/Yox1 target genes in an *srb2Δ yhp1-13A yox1-9A* triple mutant compared to the individual mutants (S4A–S4C Fig). These data suggest that when mutations in RNA Polymerase II regulators are combined with *yhp1-13A yox1-9A* they cause a more severe growth defect by further decreasing the expression of essential Yhp1/Yox1 target genes. Interestingly, negative genetic interactions were also identified between *yhp1Δ yox1Δ* and genes that regulate transcription, including *SRB2* (Figs 1G and S4D). Since Yhp1/Yox1 target genes are de-repressed in the *yhp1Δ yox1Δ* strain, this negative interaction must occur through an alternative mechanism. However, since widespread cellular processes are likely affected when Pol II-mediated transcription is reduced, it is not surprising that Pol II-associated mutations display negative interactions with both gain of function and loss of function Yhp1/Yox1 mutations.

### Blocking Yhp1/Yox1 phosphorylation improves fitness of condensin mutants

One striking finding from the SGA screens was that mitotic genes were enriched among the genes that display positive interactions with *yhp1-13A yox1-9A*, and among the genes that display negative interactions with *yhp1Δ yox1Δ* (Fig 1H). We hypothesized that these interactions may be related to the increase in fitness and resistance to microtubule disruption that is observed in *yhp1-13A yox1-9A* strains (Fig 1A and 1B) and therefore investigated these interactions further.

**Fig 4:**
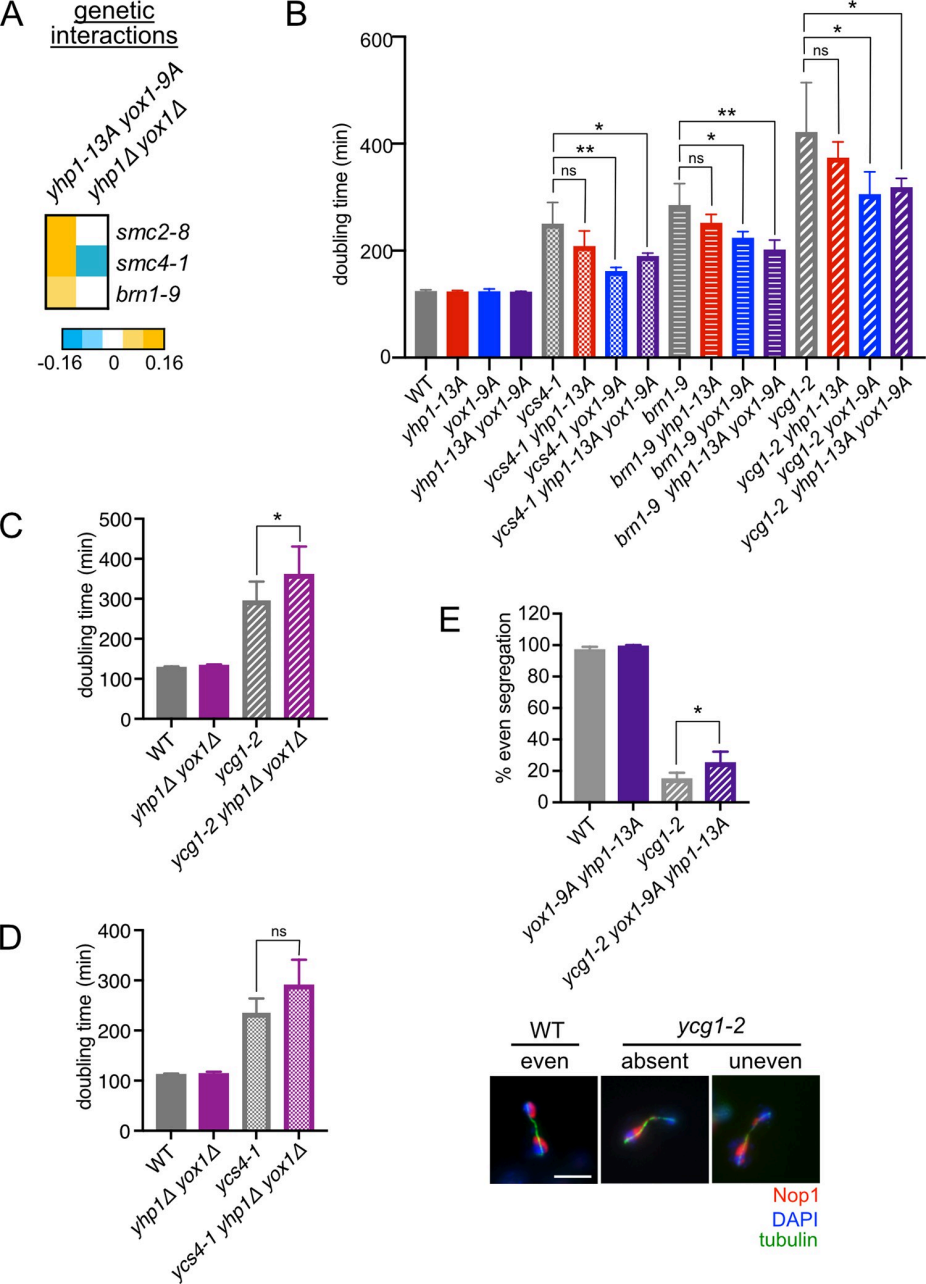
Yhp1/Yox1 phosphomutants improve proliferation of condensin mutant strains. **(A)** Genetic interactions between double mutant query strains and conditional alleles of condensin genes, from SGA screens described in Fig 1. **(B)** Doubling times of strains with the indicated genotypes grown in rich media at 28°C. Shown are an average of n = 3–6 replicates, error bars indicate standard deviations. **(C)** Average doubling times of the indicated strains grown at 28°C for n = 4–10 replicates, error bars indicate standard deviations. **(D)** Average doubling times of the indicated strains grown at 30°C for n = 3–7 replicates, error bars indicate standard deviations. For parts (B-D) significance was tested using an unpaired t-test, *p < 0.05, **p < 0.005, ns = nonsignificant. **(E)** Quantitation of nucleolar segregation in the indicated strains. Nop1 segregation was scored in at least 100 cells in n = 3 replicate experiments. Shown is average percentage of even Nop1 segregation, error bars indicate standard deviations. Significance was tested using a paired t = test, *p < 0.05. Representative images are shown comparing even segregation in a wild type (WT) strain to absent and uneven segregation that occur most frequently in *ycg1-2* strains. Scale bar represents 5 μM.

Among the genes that displayed positive interactions with *yhp1-13A yox1-9A* were three of the five subunits of the condensin complex (Figs 1H and 4A), which is essential for controlling chromosome structure during mitosis. Doubling time assays confirmed that *yhp1-13A yox1-9A*, and to a lesser extent each single mutant, partially reversed the slow growth phenotype of *brn1-9* cells at the semipermissive temperature (Fig 4B). Similar rescue was observed in strains bearing temperature-sensitive alleles of *YCG1* and *YCS4*, the two condensin subunits that were not identified in the screen. In addition, *yhp1-13A yox1-9A* increased the proliferation rate of cells expressing reduced levels of Smc4 mediated by an *SMC4-AID* (auxin-inducible degron) allele (S5A and S5B Fig), confirming the positive interaction between *yhp1-13A yox1-9A* and condensin when condensin activity is reduced by an alternate mechanism. Notably, deletion of *YHP1* and *YOX1* had the opposite effect on condensin mutants. The *yhp1Δ yox1Δ* screen identified a negative interaction with *smc4-1* (Fig 4A), and *yhp1Δ yox1Δ* reduced the proliferation rate of *ycg1-2* and *ycs4-1* strains when these mutants were examined directly, although the delay in *ycs4-1* was not statistically significant (Fig 4C and 4D).

Condensin complexes act throughout the genome to compact chromosome structure and enable accurate chromosome segregation [25–27]. An essential function of the complex in budding yeast is to mediate the compaction of the repetitive rDNA locus on chromosome XII, which is necessary for nucleolar segregation [28–30]. Although *yhp1-13A yox1-9A* only partially rescues proliferation defects in condensin mutants (Figs 4B and S5), we asked whether these mutations had any effect on nucleolar segregation that might explain the partial rescue. To test this, segregation of the nucleolar marker Nop1 was assayed in *ycg1-2* mutant cells with or without *yhp1-13A yox1-9A*. Cells were arrested in G1 at the permissive temperature and then released into the cell cycle at 30°C, which is the restrictive temperature for *ycg1-2*. Consistent with previous reports [31,32], *ycg1* mutants displayed a severe nucleolar segregation defect, with only 15% of cells evenly segregating their nucleoli (Fig 4E). However, *ycg1-2 yhp1-13A yox1-9A* cells showed even nucleolar segregation in 25% of cells, demonstrating a partial rescue of this essential condensin function.

### Yhp1/Yox1 phosphorylation establishes the lengths of cell cycle phases

**Fig 5:**
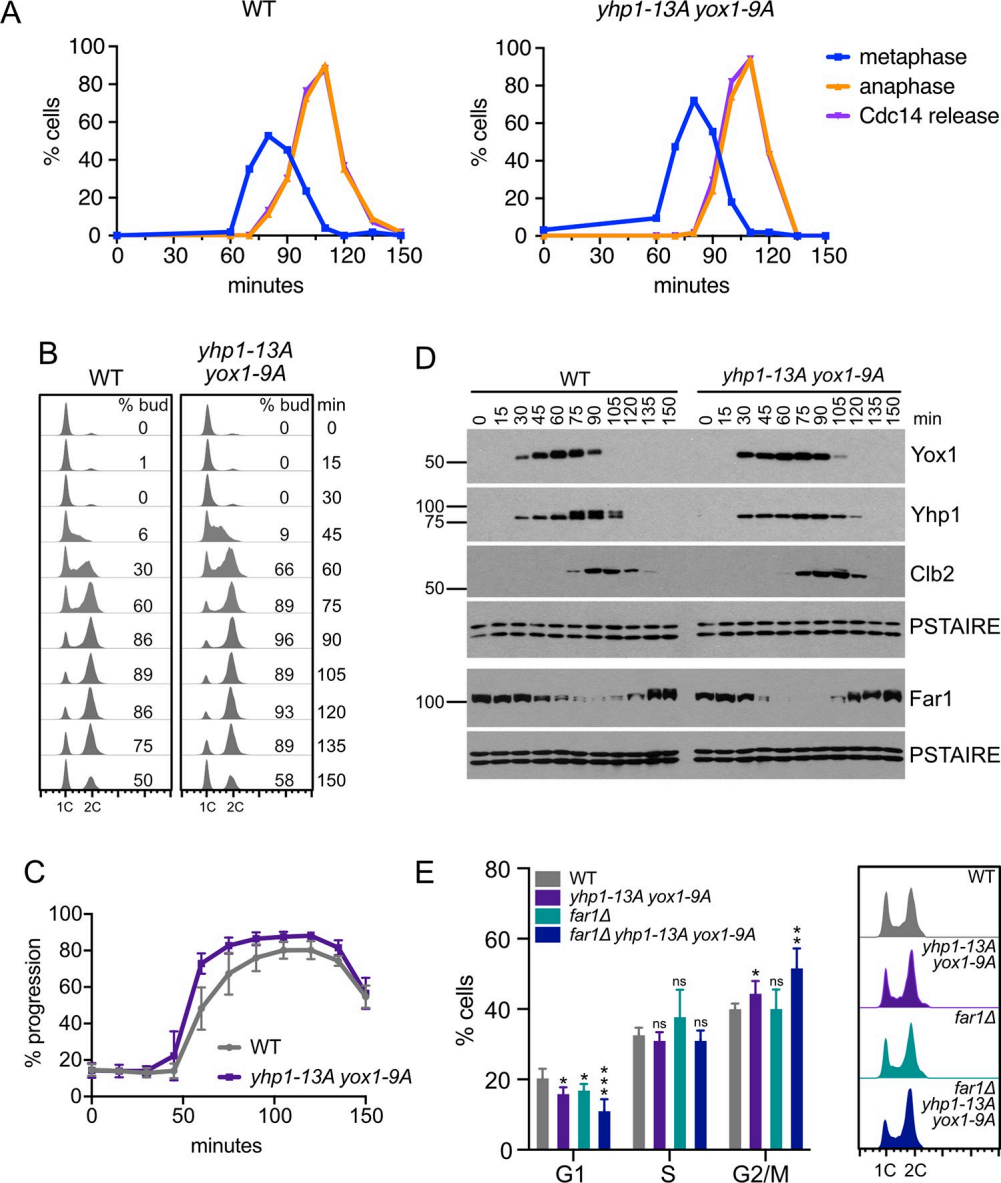
Altered cell cycle timing in *yhp1-13A yox1-9A* cells. **(A)** Cells were arrested in G1 with alpha factor and mitotic progression was quantified in the indicated strains following release from arrest at 23°C. Percentage of metaphase and anaphase spindles are shown, as well as the percentage of cells in which Cdc14 has been released from the nucleolus. A representative experiment is shown. See S6 Fig for mean percentage of metaphase spindles at the 60-minute time point from n = 3 experiments. **(B)** DNA content of the indicated strains following release from a G1 arrest at 23°C, as in (A). Percentage of budded cells at each time point is indicated. Shown is a representative experiment. **(C)** Quantitation of DNA content as a proxy for cell cycle progression. Shown are average values from n = 3 experiments performed as described in (A), error bars indicate standard deviations. **(D)** Western blots of 3V5-tagged Yox1/Yox1-9A, 13Myc-tagged Yhp1/Yhp1-13A, Clb2 and 3V5-tagged Far1 following release from G1 arrest at 23°C. PSTAIRE blots are shown as loading controls. **(E)** DNA content was measured by flow cytometry in the indicated strains growing asynchronously at 23°C. Average percentage of cells in each phase is shown in the graph at the left, representative plots are shown at the right. Graph shows an average of n = 6 replicates, errors bars indicate standard deviations. Significance of the changes in each genotype compared to WT were determined by repeated measures two-way ANOVA with Geisser-Greenhouse correction and Holm-Šídák’s multiple comparisons test, *p < 0.05, **p < 0.01, ***p < 0.005, ns = non-significant.

Having established that *yhp1-13A yox1-9A* partially rescues rDNA segregation and growth defects in cells with condensin mutations, we sought to identify the mechanism of this rescue. Importantly, expression of condensin genes was unchanged in RNA-seq experiments (Fig 2G and S5 and S6 Datasets), ruling out the possibility that *yhp1-13A yox1-9A* simply increased the expression of hypomorphic condensin alleles. Since Yhp1/Yox1 are repressors of mitotic gene expression, we considered the possibility that reducing expression of mitotic genes could lengthen mitosis, providing cells more time to accurately segregate their chromosomes. However, the cell cycle is not notably lengthened in *yhp1-13A yox1-9A* cells (Fig 1A), which argues against this possibility. To test this directly, we compared progression through mitosis between wild type and *yhp1-13A yox1-9A* cells. Cells were synchronized in G1 phase and mitotic progression was followed by examining mitotic spindle morphology and release of the phosphatase Cdc14 from the nucleolus, which is an established marker of anaphase [33]. These experiments suggested that the timing of anaphase was nearly identical in the two strains, peaking at 110 minutes after release from G1 (orange and purple lines, Fig 5A). Although this result suggested that there was no effect on mitotic timing in the mutant, we reproducibly observed that at the 60-minute time point the *yhp1-13A yox1-9A* mutant displayed an increased percentage of metaphase spindles compared to wild type cells at the same time point (blue lines, Figs 5A and S6). This suggested that *yhp1-13A yox1-9A* cells may start metaphase earlier than wild type, which would equate to a lengthening of mitosis.

To examine this possibility, we followed progression through early stages of the cell cycle in the two strains. Surprisingly, *yhp1-13A yox1-9A* strains increased budding and started DNA replication earlier than wild type, indicating that the G1/S transition was accelerated (Fig 5B–5C). Additionally, the mitotic cyclin Clb2 increased in expression earlier in the phosphomutant compared to wild type (Fig 5D, compare 60-75-minute time points), consistent with an earlier entry into metaphase. Since *yhp1-13A yox1-9A* cells enter mitosis earlier than wild type cells, but reach anaphase at the same time, these findings support our hypothesis that *yhp1-13A yox1-9A* cells spend more time in mitosis. To investigate this possibility in an alternate way, we also examined the cell cycle in asynchronously growing cells using flow cytometry. Notably, phosphomutants had a reduced percentage of cells in G1 and an increased percentage of cells in G2/M, compared to wild type (Fig 5E). This further demonstrates that *yhp1-13A yox1-9A* cells display a redistribution of cell cycle timing, they spend less time in G1 and more time in mitosis.

We next investigated how the G1/S transition was accelerated in *yhp1-13A yox1-9A* cells. We examined expression of all known G1/S regulators in our list of Yhp1/Yox1-regulated genes to determine if any genes known to inhibit S-phase entry could be Yhp1/Yox1 targets whose expression are reduced in the mutant. The only established G1/S regulator that was included on the list of Yhp1/Yox1 targets was the Cdk1 inhibitor Far1. Far1 is transcriptionally induced when cells are exposed to mating pheromone, when it functions to arrest cells at the G1/S transition to facilitate mating [34]. However, even in the absence of mating pheromone, *FAR1* is a cell-cycle regulated gene [1,35], and its deletion accelerates the G1/S transition [36,37]. We compared Far1 expression in wild type and *yhp1-13A yox1-9A* cells after release from G1 and found that Far1 protein levels decreased earlier in *yhp1-13A yox1-9A* cells (Fig 5D), and that the decrease in Far1 correlated with the entry into S phase (Fig 5B–5C). To test whether decreased Far1 could explain the accelerated S phase that we observed, we deleted *FAR1* in wild type and *yhp1-13A yox1-9A* strains. Similar to *yhp1-13A yox1-9A* strains, *far1Δ* decreased the percentage of cells in G1 phase (Fig 5E). However, there was an additive effect when these mutations were combined, with an even greater decrease in the percentage of G1 cells in *yhp1-13A yox1-9A far1Δ* strains. This result suggests that although *FAR1* may be a Yhp1/Yox1 regulated gene, its downregulation is not likely to be responsible for the acceleration S phase entry in *yhp1-13A yox1-9A* cells. Therefore, this effect must be mediated by additional Yhp1/Yox1 target genes.

### Increased repression of mitotic exit genes improves the fitness of condensin mutants

We next sought to identify the Yhp1/Yox1 target genes whose downregulation causes the observed delay in mitotic progression and rescues the proliferation defect in condensin mutants.

**Fig 6:**
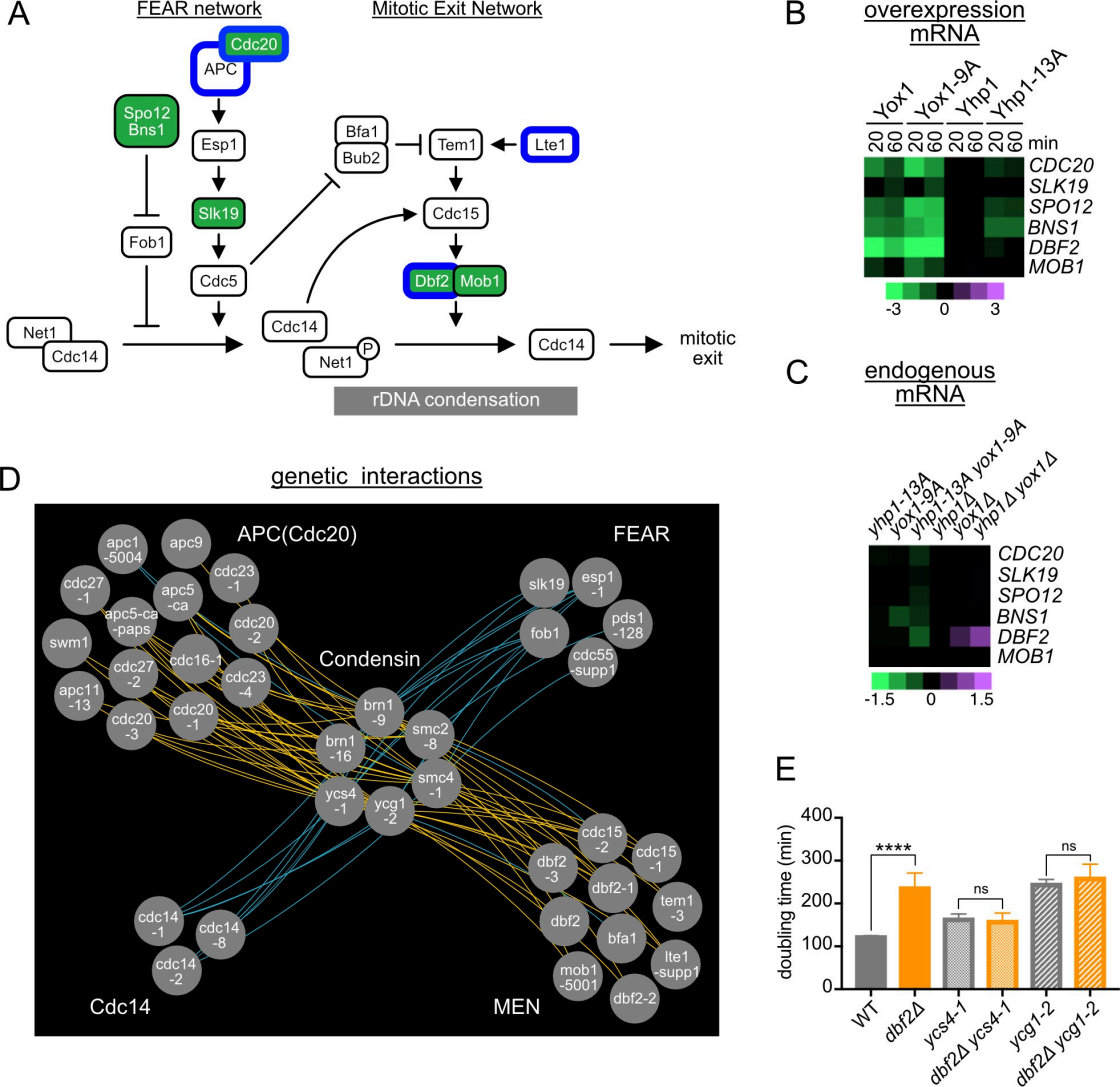
Increased repression of mitotic exit genes by *yhp1-13A yox1-9A* improves the fitness of condensin mutants. **(A)** Diagram of the FEAR and MEN pathways that control mitotic exit. Regulators colored green are repressed by Yhp1/Yox1. Regulators circled in blue display negative genetic interactions with *yhp1-13A yox1-9A*. **(B)** Gene expression of Yhp1/Yox1 target genes that regulate mitotic exit, following acute overexpression of the indicated proteins, from RNAseq experiments presented in Fig 2. **(C)** Expression of genes from (B) in strains expressing the indicated Yhp1/Yox1 mutants from their endogenous promoters or deleted for the indicated TFs, from experiments presented in S2 Fig. Note the difference in scale between (B) and (C). **(D)** Genetic interaction map highlighting significant genetic interactions between condensin and mitotic exit regulators. Yellow lines indicate positive interactions, blue lines indicate negative interactions. All interaction values are included in S1 Table. **(E)** Doubling times of strains with the indicated genotypes grown in rich medium at 28°C. Shown is an average of n = 7–15 replicates, error bars indicate standard deviations. Significance was tested with an unpaired t-test, ****p < 0.0001, ns = nonsignificant.

Condensin-mediated rDNA condensation is coordinated with the steps of mitotic exit, which is regulated by two connected pathways, FEAR (Fourteen Early Anaphase Release) and MEN (Mitotic Exit Network). The FEAR pathway triggers partial release of the phosphatase Cdc14 from the nucleolus and is required for condensin recruitment to the rDNA and accurate rDNA segregation [31,38]. Condensin then remains active until Cdc14 is fully released from the nucleus, through the action of the MEN pathway (Fig 6A). Mutations in the MEN pathway prolong the period of condensin-mediated rDNA compaction [39]. Notably, genes in these pathways were highly enriched among Yhp1/Yox1 target genes (Figs 2G and 6A–6C). In addition, negative genetic interactions were identified between *yhp1-13A yox1-9A* and several genes in these pathways, including *dbf2* (Figs 1H–3D and 6A). These results suggested that *yhp1-13A yox1-9A* cells express reduced levels of FEAR/MEN genes, which may prolong the period of condensin activity during mitosis, partially reversing the rDNA segregation and proliferation defects in condensin mutant cells.

If this hypothesis is correct, the expectation is that mutations in FEAR and/or MEN genes should, like *yhp1-13A yox1-*9A, display positive genetic interactions with condensin mutants. To test this, we examined previously reported genetic interactions among all condensin alleles and FEAR/MEN regulatory genes [19]. Consistent with our prediction, condensin alleles displayed positive genetic interactions with 23% of FEAR/MEN regulatory genes, but only 3% of all yeast genes, for a 7.34-fold enrichment (p-value < 1e^-16^). Upon closer examination, almost all strains with mutations in the MEN displayed positive interactions with condensin alleles (Fig 6D and S1 Table). To support these data, we directly tested for genetic interactions between *dbf2Δ* and two condensin alleles, *ycg1-2* and *ycs4-1* (Fig 6E). We found that while *dbf2Δ* significantly increased the doubling time of wild type cells, it did not significantly change the doubling times of condensin mutants, confirming the positive interactions. Notably, among MEN regulators *bfa1Δ* was an exception and showed a negative genetic interaction with *ycg1-2* (Fig 6D). However, this interaction is consistent with our model, since Bfa1 is a negative regulator of the MEN [40]. Positive interactions also occurred between condensin and the Anaphase Promoting Complex (APC), which both triggers the metaphase-anaphase transition and initiates the MEN/FEAR pathways through the activation of separase [41]. In contrast, condensin mutations displayed negative interactions with mutations in FEAR genes and alleles of *CDC14*, consistent with previous reports that these genes are required for condensin function [31,38]. Together, these results support the model that blocking phosphorylation of Yhp1/Yox1 enhances proliferation in cells with compromised condensin function by reducing expression of mitotic exit genes, delaying mitotic exit, and allowing more time for the partially functional condensin complex to condense the rDNA and accurately segregate the genome.

## Discussion

**Fig 7:**
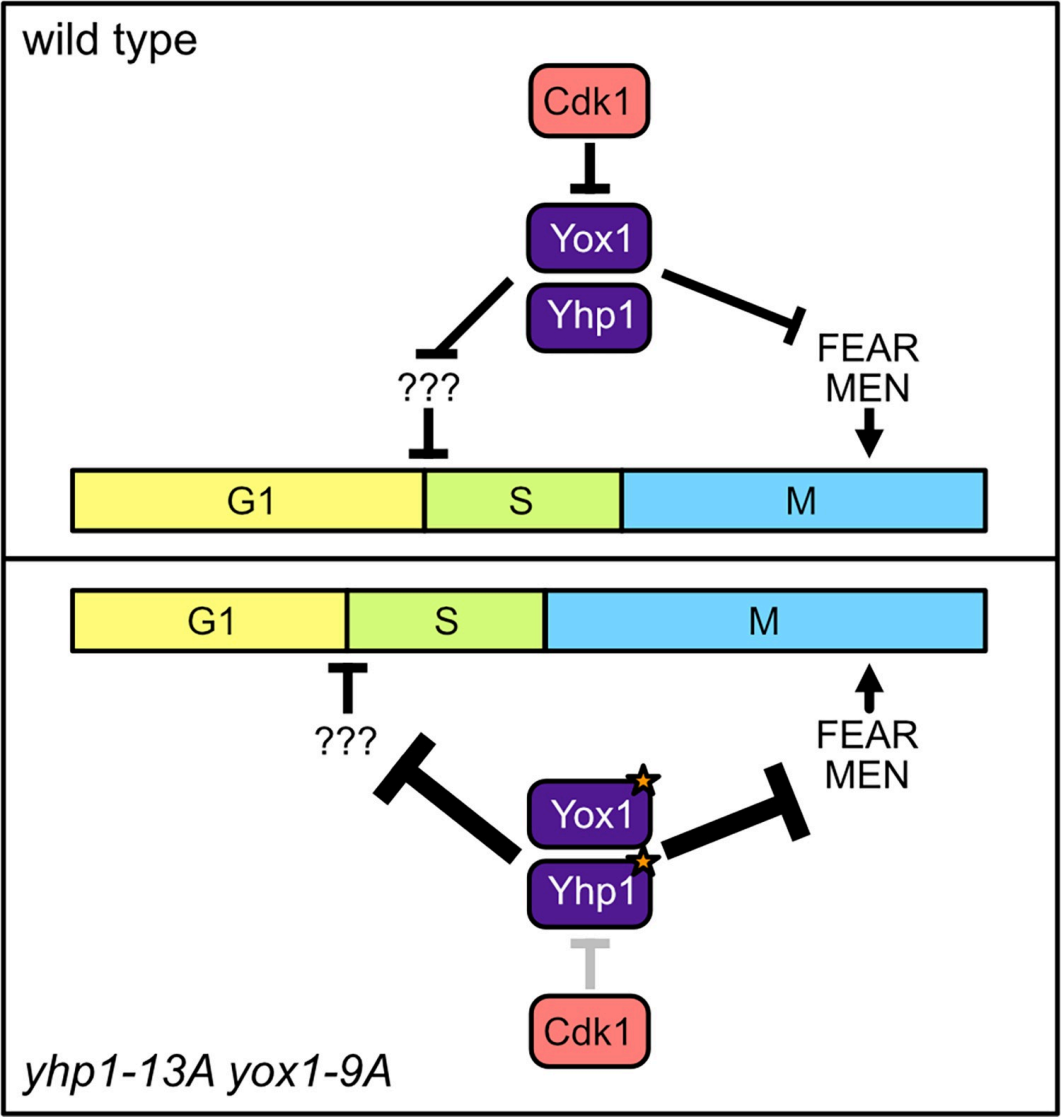
Model of cell cycle regulation by Yox1 and Yhp1.

Here, we examined how phosphorylation events that have seemingly little effect on the cell cycle affect cellular physiology. Our data suggests that Cdk1 acts through Yhp1/Yox1 to establish the relative lengths of cell cycle phases. During a normal cell cycle, *YOX1* is expressed as cells enter S phase and *YHP1* is expressed slightly later [12]. Upon their expression, Yox1 and Yhp1 redundantly repress expression of many genes that are required to initiate S phase, including genes that control the timing the G1/S transition (Fig 7). Yhp1/Yox1 also repress expression of mitotic genes, preventing their premature expression and controlling the timing of mitosis. A key component of Yhp1/Yox1 regulation is their phosphorylation-mediated degradation by the ubiquitin proteasome system. Concomitant with the expression of Yhp1/Yox1, Cdk1 activity increases, which results in both TFs being targeted for degradation and limiting their expression [12]. As a result, blocking Yhp1/Yox1 phosphorylation results in their elevated expression at the first point in the cell cycle in which they are detected. This likely causes decreased expression of one or more genes that controls the G1/S transition and as a result, accelerates entry into S-phase. Blocking phosphorylation also lengthens the period in which Yhp1/Yox1 are expressed, resulting in a decrease in expression of mitotic exit genes and a lengthening of mitosis.

These cell cycle effects were not identified previously, most likely because standard assays that measure proliferation show no apparent effect on overall cell cycle length in these mutants (Fig 1A) [12]. However, we observe a proliferative advantage in mutant cells when they are competed against wild type cells in a co-culture assay (Fig 1B). This increase in fitness is a surprising result, and it raises the question of why phosphoregulation of Yhp1/Yox1 has evolved if it normally limits proliferation. A likely possibility is that blocking of phosphorylation of Yhp1/Yox1 enhances proliferation when cells are grown in optimal conditions, but results in a growth disadvantage in other environments. In support of this possibility, we found that *yhp1-13A yox1-9A* cells display increased sensitivity to cell wall stressors (S7 Fig). This observation can explain why phosphoregulation of these TFs has been retained over evolution.

Our gene expression data suggested that the G1/S acceleration in phosphomutant cells might be due to increased repression of the Cdk1 inhibitor Far1, since it is the only established G1/S inhibitor that was repressed by Yhp1/Yox1. We confirmed earlier findings that suggested deletion of *FAR1* accelerates the G1/S transition [36,37]. However, the shortening of G1 that we observed was additive when combined with *yhp1-13A yox1-9A* (Fig 5E), indicating that Yhp1/Yox1 do not control G1/S timing via repression of *FAR1*. Interestingly, an accelerated G1/S transition is also observed in *yhp1Δ yox1Δ* strains [18]. This result is puzzling since the expectation is that phosphomutant and deletion strains would have opposite effects. This could be due to differences in experimental setup. Or alternatively, the G1/S acceleration might occur in both strains, but by different mechanisms. Additional study will be required to answer this question and understand the mechanistic basis of G1/S acceleration in *yhp1 yox1* mutants.

Notably, many essential DNA replication regulators, such as the *MCM2-7* genes, are also repressed by Yhp1/Yox1. The fact that phosphomutant cells do not exhibit an S-phase delay suggests transcription of these genes is not limiting for DNA replication. Expression of the *MCM2-7* genes peaks at the M/G1 transition and the proteins are very stable [42], so their repression by Yhp1/Yox1 in S-phase may serve as a backup mechanism to ensure new proteins are not synthesized, and that replication can no longer be initiated as cells enter mitosis.

Importantly, although G1/S and metaphase are accelerated in *yhp1-13A yox1-9A* cells compared to wild type, anaphase occurs at the same time relative to G1 release, which equates to a lengthening of mitosis (Fig 5A). Our findings support the model that it is this mitotic delay that improves rDNA segregation and the fitness of condensin mutants. Because many genes controlling mitotic exit are Yhp1/Yox1 targets, it is likely that the combined reduction in expression of several genes delays mitotic exit. This possibility is also supported by the finding that partially functional alleles of every known positive regulator in the MEN displays positive genetic interactions with condensin (Fig 6D). However, *DBF2* is one of the most strongly downregulated genes in *yhp1-13A yox1-9A* cells, so it is possible that it may be a larger contributor to the phenotype. An alternative possibility is that the delay in mitosis is not due to downregulation of mitotic genes but is indirect and the result of DNA damage checkpoint activation in response to the accelerated S-phase entry. However, we saw no evidence of Rad53 activation in *yhp1-13A yox1-9A* cells (S8 Fig). A likely possibility is that the acceleration of S-phase that occurs in *yhp1-13A yox1-9A* cells is not great enough to lead to problems with DNA replication that can cause DNA damage.

We chose to focus on the mechanism of rescue of condensin mutants, since they were highly overrepresented among *yhp1-13A yox1-9A* positive interacting genes. However, the lengthening of mitosis is likely to explain other identified positive interactions, as well as the increased resistance to microtubule poisons that is observed in *yhp1-13A yox1-9A* mutants (Fig 1A). Providing more time for mitosis to be completed could rescue diverse chromosome segregation issues that arise in cells, and therefore increase overall fitness. Previous studies showing that genome stability is improved by mutations that slow the cell cycle supports this model [43–45]. However, in contrast to these examples, the overall rate of proliferation is increased in Yhp1/Yox1 phosphomutants. Our results demonstrate how mutations that change the distribution of cell cycle phases increase overall fitness.

Previous analysis of the global genetic interaction network revealed that cell cycle mutants account for 30% of positive interactions among essential genes [19]. This discovery led to speculation that cell cycle mutants exhibit increased numbers of positive interactions because slowing down the cell cycle gives cells more time to deal with compromised cellular processes. Our findings that reveal the mechanism of condensin rescue provide experimental evidence to support this model. In addition, since *yhp1-13A yox1-9A* specifically affects mitotic timing, the spectrum of positive interacting genes may reflect specific defects that are improved by slowing down mitosis. This not only includes mutations in genes directly involved in mitotic progression, but other cellular processes such as ribosome biogenesis and ncRNA/rRNA processing, which are enriched among the positive interactions. Conversely, the spectrum of negative interacting genes reveals the cost of increasing Yhp1/Yox1 activity. For example, *yhp1-13A yox1-9A* cells may be more susceptible to fluctuations in transcription or energy availability, since they exhibit negative interactions with genes that regulate transcription and chromatin, as well as those regulating mitochondrial function.

One surprising finding from the Yhp1/Yox1 genetic interaction screens is that gain of function (*yhp1-13A yox1-9A*) and loss of function (*yhp1Δ yox1Δ*) mutations exhibited many shared categories of genetic interactions. At the outset, the prediction was that opposite interactions might occur; for example, positive interactions with a group of genes in the phosphomutant and negative interactions in the deletion. Although this pattern was observed for a list of curated mitotic genes (Fig 1H), the direction of the interactions was the same for most categories that were enriched in SAFE and GO term enrichment analyses. This may occur because the categories of genes that exhibit this behavior are expected to have pleiotropic effects. For instance, mutations that impact RNA Pol II-mediated transcription, mitochondrial function or translation are expected to influence multiple, unrelated cellular processes. It is also the case that since the categories identified by SAFE and GO term analysis are very broad, some genes within a given category may exhibit opposite interactions despite the common enrichment of the process. For example, the condensin allele *smc4-1* showed a positive interaction with *yhp1-13A yox1-9A* and a negative interaction with *yhp1Δ yox1Δ*.

Finally, our work has implications for understanding how mutations in cell cycle genes affect the proliferation of cancer cells. Although Yox1 and Yhp1 do not have direct homologs in the cell cycle-regulatory TF network in humans, Cdk phosphorylation similarly controls the activities of transcriptional repressors and activators to drive gene expression in the correct cell cycle stages. Mutations that affect the activities of cell cycle TFs and reduce expression of mitotic genes may lead to similar effects on chromosome segregation and cellular fitness as the mutations we have investigated here. Moreover, as the availability of whole genome sequencing of tumors increases, more mutations are being identified in cell cycle-regulatory genes. Our findings suggest that mutations that arise in cancer cells may not affect proliferation in a predictable manner, even if there is a good understanding of the molecular function of the mutated genes. Further investigation into the network-wide consequences of cell cycle mutations should shed light on this possibility.

## Materials and methods

### Yeast strains and growth conditions

All strains are in the BY4741 background and listed in S2 Table. Genetic manipulations were carried out using standard methods [46,47]. Strains were grown in rich medium (YM-1) or synthetic complete medium (C) with 2% dextrose at 30°C, unless otherwise indicated. To construct strains bearing estradiol-inducible Yhp1/Yox1 proteins, PCR products including a C-terminal portion of *YHP1* or *YOX1*, with or without mutations in Cdk1 sites, and a C-terminal 3V5 tag were transformed into previously described strains (EH1456 and EH2877) with the Z3EV promoter integrated upstream of each gene [24]. Matched control strains were constructed by transforming the same PCR products into a strain with the Z3EV promoter integrated upstream of the non-functional *HO* gene (EH2407).

Marker strains for competition assays were constructed by recombining a GFP expression cassette (*Hyg-TEF1p-GFP*) into a gene-free locus on chromosome VI. To construct a matched negative control strain, a previously characterized point mutant of GFP that is non-fluorescent was used in place of wild type GFP (GFP-Y66F) [48]. To generate strains for analysis, these GFP+ and GFP- marker strains were crossed to independently derived strains carrying phosphosite mutations in *YHP1* and *YOX1*, and progeny were isolated that carried both phosphomutant alleles and the desired GFP marker. Wild type strains that were competed against phosphomutant strains were generated by a similar mechanism: strains carrying identically tagged alleles of wild type *YHP1* and *YOX1* were crossed to GFP marker strains to obtain progeny with tags on both *YHP1* and *YOX1* and expressing the desired GFP marker. A list of all strain isolates that were generated and tested is included in S1A Fig.

### Doubling time assays

Cultures were grown to logarithmic phase, diluted to 0.1 optical densities, and aliquoted in triplicate into a 96-well plate. Cultures were then incubated at the temperature indicated in each figure legend, with shaking, in a Tecan Infinite M200 Pro or Infinite M Nano plate reader. Optical densities at 600 nM were measured every 20 minutes until cultures reached approximately 0.8 optical densities. Doubling times were calculated by fitting data points between 0.2 and 0.5 optical densities to an exponential growth equation using GraphPad Prism software. Whenever possible, multiple isolates of strains with each genotype were assayed and included in the analysis. Samples were excluded from the analysis if they failed to grow in the plate reader following dilution, which occurred sporadically with temperature sensitive strains.

### Competition assays

Cells with fluorescent and non-fluorescent GFP (GFP-Y66F) were mixed in equal proportions in 10 mL synthetic complete medium with 2% dextrose. Immediately following mixing, 0.15 optical densities were collected, pelleted by centrifugation, resuspended in 2 mL sodium citrate buffer (50 mM sodium citrate, 0.02% NaN_3_, pH 7.4), and stored at 4°C for analysis by flow cytometry. All mixed cultures were then diluted to 0.004 optical densities in 10 mL and incubated at 30°C. Every 12 hours thereafter cultures were sampled as above and diluted to 0.004–0.008 optical densities to ensure that the population did not exceed logarithmic growth for the duration of the experiment (72 hours). Percentage of GFP-positive cells in each sample was quantified using a Guava EasyCyte HT flow cytometer and analyzed with FlowJo software.

### SGA screens and functional enrichment analyses

SGA experiments and selection steps were performed as previously described [19,49]. We used an intermediate genetic interaction score cutoff (genetic interaction score, |ε| ≥ 0.08, P < 0.05) [19] to identify genes that interact with the Yhp1/Yox1 double mutant (S1 Dataset). From the *yhp1-13A yox1-9A* phosphomutant SGA screen, we found negative interactions with 178 genes (nonessential: 128, essential: 50) and positive interactions with 122 genes (nonessential: 82, essential: 40). From the *yhp1Δ yox1Δ* double deletion SGA screen, we found negative interactions with 555 genes (nonessential: 420, essential: 135) and positive interactions with 562 genes (nonessential: 404, essential: 158).

We analyzed the functional coherence of the positive and negative interacting genes using SAFE (spatial analysis of functional enrichment) analysis, GO—bioprocess enrichment analysis, and enrichment analysis on manually curated cell cycle gene-sets. Negative and positive interactions were analyzed separately for each screen.

The purpose of our SAFE analysis [50] was to find which functional regions on the global genetic interaction network [19] (Fig 1E) are enriched for a given list of positive or negative interacting genes from our double mutant screens. First, we overlapped the genes from the nonessential deletion mutant array and temperature-sensitive essential array with the genes present on the global genetic interaction network [19] and used these genes as the background for enrichment analysis. Next, we used the SAFE method to evaluate the enrichment of each gene on the global genetic interaction network for the provided genes in its neighborhood, assessed by the hypergeometric distribution. All enriched genes (p < 0.05) on the global genetic interaction map are plotted in blue (negative interactions) and yellow (positive interactions). Then we highlighted those functional regions that have more than expected enriched genes (p < 0.05). SAFE analysis for the *yhp1-13A yox1-9A* genetic interaction screen is presented in Fig 1F and S2 Dataset. SAFE analysis for the *yhp1Δ yox1Δ* genetic interaction screen is presented in Fig 1G and S3 Dataset.

We evaluated if the positive/negative interactions from the *yhp1-13A yox1-9A* and *yhp1Δ yox1Δ* screens were associated with any GO processes with at least 5 genes and at most 500 genes. The significance of enrichment of GO processes was measured with the hypergeometric distribution. All GO processes with enrichment p < 0.05 can be found in S2 Dataset for *yhp1-13A yox1-9A* associated genetic interactions and in S3 Dataset for *yhp1Δ yox1Δ* associated genetic interactions.

We also tested the functional enrichment of Yox1/Yhp1-associated genetic interactions using four manually curated cell cycle gene-sets: (1) cell cycle s288c genes (632 genes); (2) cell cycle s288c mitosis specific genes (90 genes); (3) mitotic exit associated genes (32 genes); (4) condensin genes (5 genes). Lists of genes and descriptions of each group are included in S4 Dataset. Corresponding analysis can be found in S2 Dataset for *yhp1-13A yox1-9A* associated genetic interactions and in S3 Dataset for *yhp1Δ yox1Δ* associated genetic interactions (Fig 1H).

### Western blotting

Lysates for Western blotting were prepared using TCA extraction, as previously described [51]. Following SDS-PAGE and transfer to nitrocellulose, blots were probed with antibodies against PSTAIRE (P7962, Sigma), V5 (R96025, Invitrogen), MYC (clone 9E10, M5546, Sigma), Clb2 (y-180, Santa Cruz Biotechnology), Rad53 (ab104232, Abcam) or G6PDH (A9521, Sigma) as indicated.

### RNA-seq and data analysis

For all RNAseq experiments, RNA was purified from five optical densities of cells using an acid-phenol purification as previously described [52]. Cells with estradiol-inducible wild type or phosphomutant Yox1 or Yhp1 and respective controls were grown in YM-1 to mid log phase before the addition of estradiol to a final concentration of 1 μM. Samples were collected prior to estradiol addition (t = 0), as well as 20- and 60-minutes following estradiol addition. Poly-A purification, library construction, and sequencing were performed by BGI Americas. All sequencing data is available from NCBI-GEO with accession number GSE188180.

RNA-seq analysis was performed using OneStopRNAseq [53]. Specifically, FastQC and MultiQC [54] were used for raw read quality control. Paired-end reads were aligned to Saccharomyces_cerevisiae.R64-1-1, with star_2.5.3a [55], annotated with Saccharomyces_cerevisiae.R64-1-1.90.gtf. Aligned exon fragments with mapping quality higher than 20 were counted toward gene expression with featureCounts_1.5.2 [56]. Differential expression (DE) analysis was performed with DESeq2 [57]. Within DE analysis, ’ashr’ was used to create log2 Fold Change (LFC) shrinkage for each comparison. For experiments with strains constitutively expressing *YHP1* and *YOX1* mutations or deletions, genes were considered significantly changed if FDR < 0.1 (S2 Fig and S5 Dataset) [58]. For strains expressing inducible *YHP1* or *YOX1* genes and matched controls, LFC was calculated at 20- and 60-minutes compared to t = 0 for each strain. These LFC values in control strains were then subtracted from matched experimental strains to create background subtracted LFC values and eliminate any changes that may have resulted from estradiol treatment independent of expression of Yox1/Yhp1 proteins. For these experiments, genes were considered significantly changed if background subtracted LFC values were > |0.58| (1.5-fold) and had an FDR < 0.05. Background-subtracted LFC values for genes that fit these criteria are plotted in Fig 2B and data is included in S6 Dataset.

For genes whose expression values are significantly changed upon acute overexpression of Yhp1, Yhp1-13A, Yox1, or Yox1-9A, we repeated the same set of enrichment analyses described above (see SGA screens and functional enrichment analyses) and these enrichment results can be found in Fig 2F and 2G and S7 Dataset. To identify TF binding motifs in promoters of these genes, PFM motif files were downloaded from YeTFaSCo [59]. Expert curated motifs were used. FIMO [60] was then employed to find motif matches in the promoter regions (500bp upstream TSS) of 332 regulated genes, and 3320 random 500bp regions from *S*. *cerevisiae* genome. A Chi-squared test was used to test for motif enrichment in the promoter regions. Enrichment statistics and motif matches can be found in S8 Dataset.

### RT-qPCR

Since deletion of *SRB2* was expected to affect all Pol II-mediated transcription, a defined number of *S*. *pombe* cells was added to each sample prior to RNA purification to enable normalization. *S*. *cerevisiae* cells were grown in rich medium to mid log phase, arrested in G1 by the addition of 10 μg/ml alpha-factor for three hours (with a second equal amount added after two hours), then released into media without alpha-factor. Cells were collected 60-minutes after release and cell concentration was determined using a Guava EasyCyte HT flow cytometer. Samples were normalized for cell number such that an equivalent number of *S*. *pombe* cells (2.5e^6^) was added to 4.5e^7^ *S*. *cerevisiae* cells. RNA purification, reverse transcription and quantitative PCR was performed as previously described [12,52]. Primers used for qPCR are included in S3 Table. Levels of Yhp1/Yox1 target genes were determined by normalizing values to *S*. *pombe ACT1*, and log2 fold change values were calculated with respect to mRNA levels in wild type cells.

### Nop1 segregation

Cells were grown to mid-log phase at the permissive temperature (23°C) and arrested in G1 with 10 μg/ml alpha-factor for three hours (with a second equivalent amount added after two hours). Cells were then released into medium without alpha-factor at the restrictive temperature (30°C) to inactivate *ycg1-2*. Cells were collected at 60-, 75-, and 90-minutes following release to enrich for anaphase cells. Samples were fixed and stained as previously described [61], with minor modifications. Permeabilized cells were stained with anti-tubulin (YOL1/34, Invitrogen) and anti-Nop1p primary antibodies (Encor) for two hours, and anti-rat FITC (Jackson Immunoresearch) and goat anti-mouse Alexafluor 594 (Invitrogen) secondary antibodies for two hours, both at room temperature. Cells were imaged on a Nikon Eclipse E400 microscope. To ensure that cells at similar stages of anaphase were being compared, late anaphase cells were identified as those with an elongated spindle and clearly segregated DAPI masses. Nop1 segregation was then scored as equal or unequal (either absent or uneven) in only late anaphase cells. A minimum of 100 cells per strain were scored in each experiment.

### Cell cycle progression

Cell cycle positions were determined by flow cytometry. Briefly, cells were fixed with 70% ethanol and stained with Sytox Green (Invitrogen), as previously described [61]. Samples were analyzed using a Guava EasyCyte HT flow cytometer and analyzed with FlowJo software.

To analyze progression through the cell cycle (Fig 5A–5C), cells were grown to mid-logarithmic phase at 23°C in rich medium (YM-1 with 2% dextrose), then arrested in G1 with the addition of 10 μg/ml alpha-factor for three hours (with a second equivalent amount added after two hours). Cells were then released into medium without alpha-factor and samples were collected at the indicated time points. Alpha-factor was added back after 75 minutes post-release to prevent cells from progressing into a second cell cycle. Cell cycle position was determined by flow cytometry and the number of budded cells was determined by counting a minimum of 100 cells per timepoint. Percent cell cycle progression was calculated from the mean of the DNA content in each sample, as previously described [62]. To quantify progression through mitosis, cells were fixed at timepoints between 60- and 150-minutes post-release, as indicated, and immunofluorescence performed as described above. 3HA-tagged Cdc14 was detected with anti-HA antibody (16B12, Covance). Nucleolar Cdc14 release, metaphase, and anaphase spindles were scored as previously described in a minimum of 50 cells per time point [63].

To quantify the percentage of cells in each cell cycle phase in asynchronous populations (Fig 5E), a univariate model utilizing the Watson Pragmatic algorithm [64] within the FlowJo software was employed.

### Serial dilution assays

To compare growth on agar plates, five-fold dilutions of cells were spotted onto rich or synthetic media, with the indicated additions. Plates were removed from the incubator when individual colonies were evident for the wild type (WT) strain.

## Supporting information

S1 Fig Individual replicates of all competition experiments. **(A)** Table detailing the specific GFP- and GFP+ strain isolates utilized in competition experiments. Five pairs of wild type (WT) strains were competed against each other and 10 isolates of the *yhp1-13A yox1-9A* phosphomutant (PM) were competed against the indicated WT strains. **(B)** Individual replicates of competition experiments between differentially marked WT strains. Each box includes two-three replicates for one pair of strains (defined in part A). **(C)** Replicates for each pair of strains (boxed experiments in part B) were averaged together and the average data for all 5 pairs were then averaged. Error bars represent standard deviations. **(D)** Individual replicates of competition experiments between phosphomutant (PM) and WT strains. Each box includes two-three replicates of one pair of strains (defined in part A). Average data for all 10 pairs of strains is presented in Fig 1B.
(PDF)

S2 Fig RNA-seq analysis of strains expressing *yhp1 yox1* mutations. RNA-seq data for asynchronously growing *yhp1 yox1* mutant strains. The indicated mutations are integrated at the endogenous locus of each gene. **(A)** Overlap of genes significantly downregulated in *yhp1-13A yox1-9A* and those significantly upregulated in *yhp1Δ yox1Δ*. Yhp1/Yox1 target genes should be in this category. List of genes is included in S5 Dataset. **(B)** Heat map showing log fold change of the 16 overlapping genes from part (A). **(C)** Heat map showing log2 fold change of all Yox1/Yhp1-regulated genes (defined as genes that were significantly changed upon acute overexpression of Yox1 or Yhp1, in Fig 2) in asynchronous cells with the indicated mutations integrated at each endogenous locus. Order of genes and scale matches Fig 2B.
(TIF)

S3 Fig The cell cycle is not notably changed following acute overexpression of Yhp1, Yox1 or phosphomutant proteins. Representative FACS plots showing DNA content of strains following estradiol-induced expression of Yox1/Yhp1, phosphomutant proteins, or control strains. Control for Fig 2.
(TIF)

S4 Fig Negative genetic interaction between *srb2Δ* and *yhp1 yox1* mutants. **(A)** Doubling times of the indicated strains growing at 30°C in synthetic medium. Shown is an average of n = 3–8 replicates, error bars indicate standard deviations. Significance was tested with an unpaired t-test, ** p < 0.005, ***p < 0.0005, ns = nonsignificant. **(B)** RT-qPCR of Yhp1/Yox1 target genes in the indicated strains that were synchronized in late S-phase. Shown are average log2 fold change values, compared to a wild type strain, in n = 3 experiments. Error bars indicate standard deviations. **(C)** Representative FACS plots of strains with the indicated genotypes from (B). **(D)** Doubling time of the indicated strains grown in synthetic medium at 30°C. Shown is an average of n = 3–4 replicates. Significance was tested using an unpaired t-test, *p < 0.05, ****p < 0.0001, ns = nonsignificant.
(TIF)

S5 Fig Positive genetic interactions between *yhp1-13A yox1-9A* and condensin. **(A)** Doubling times of Smc4-AID expressing strains with the indicated genotypes grown in rich medium containing 0.3 mM IAA (to degrade SMC4-AID). Shown is an average of n = 3 experiments. Significance was tested using an unpaired t-test, *p < 0.05, **p < 0.005, ns = nonsignificant. **(B)** Western blot of strains from (A) with or without the addition of 0.3mM IAA, as indicated. Smc4 is detected by a FLAG tag, TIR1 is detected by 3HA tag, PSTAIRE is shown as a loading control. **(C)** Five-fold dilutions of strains with the indicated genotypes were plated on rich medium and grown at the indicated temperatures.
(TIF)

S6 Fig Metaphase spindles arise earlier in *yhp1-13A yox1-9A* cells compared to wild type. Quantitation of metaphase spindles at the 60-minute time point following G1 arrest-release, as performed in Fig 5A. Shown in the mean percentage of metaphase spindles from n = 3 experiments. Significance was tested using a paired t-test, *p = 0.0247.
(TIFF)

S7 Fig *yhp1-13A yox1-9A* cells are sensitive to cell wall stressors. Five-fold dilutions of wild type or *yhp1-13A yox1-9A* cells were plated on synthetic complete (SC) medium, or SC medium containing 150 μg/mL Calcofluor White or 0.25 mg/mL Congo Red to elicit cell wall stress. Two isolates of each genotype are shown.
(TIF)

S8 Fig Rad53 is not phosphorylated in *yhp1-13A yox1-9A* cells following release from G1/S arrest. Cells with the indicated genotypes were arrested in G1 with alpha-factor and released into the cell cycle as in Fig 5A. Western blot of Rad53 is shown to determine if phosphorylation can be detected, which would be an indication of DNA damage. Lysates from untreated asynchronous (asy) and MMS-treated (0.05% for 3 hours) wild type cells are included as an example of a DNA-damage induced Rad53 phosphoshift. G6PDH is shown as a loading control.
(TIF)

S1 Table Genetic Interactions between condensin alleles and mitotic exit regulators. Shown are significant genetic interaction values with a p-value of < 0.05. Strong interactionswith a |ε| ≥ 0.08 are plotted in Fig 6D.
(PDF)

S2 Table Strain table. All *S*. *cerevisiae* strains are in the BY4741 background.
(PDF)

S3 Table Primer table. List of all primers used in RT-qPCR experiments.
(PDF)

S1 Dataset Primary data from SGA screens with *yhp1-13A yox1-9A* and *yhp1Δ yox1Δ*. (XLSX)

S2 Dataset Enrichment analyses of data from SGA screen with *yhp1-13A yox1-9A*. (XLSX)

S3 Dataset Enrichment analyses of data from SGA screen with *yhp1Δ yox1Δ*. (XLSX)

S4 Dataset Curated gene lists used for enrichment analyses. (XLSX)

S5 Dataset Results of RNA-seq experiments with *yhp1-13A yox1-9A*, *yhp1Δ yox1Δ* and single mutant strains. (XLSX)

S6 Dataset Results of RNA-seq experiments following acute overexpression of Yhp1, Yhp1-13A, Yox1 and Yox1-9A. (XLSX)

S7 Dataset Enrichment analyses of genes significantly changed following acute overexpression of Yhp1, Yhp1-13A, Yox1 or Yox1-9A. (XLSX)

S8 Dataset Identification of motifs for cell cycle-regulatory transcription factors in the promoters of Yhp1/Yox1-regulated genes. (XLSX)

S9 Dataset Data and statistics underlying the graphs throughout the paper. (XLSX)
