# Supplementary material for: Repression of essential cell cycle genes increases cellular fitness
Source: PLoS Genet. 2022 Aug 29;18(8):e1010349. doi: 10.1371/journal.pgen.1010349 (PMC9462756; doi:10.1371/journal.pgen.1010349)
Supplement: S1 Fig — (A) Table detailing the specific GFP- and GFP+ strain isolates utilized in competition experiments. Five pairs of wild type (WT) strains were competed against each other and 10 isolates of the yhp1-13A yox1-9A phosphomutant (PM) were competed against the indicated WT strains. (B) Individual replicates of competition experiments between differentially marked WT strains. Each box includes two-three replicates for one pair of strains (defined in part A). (C) Replicates for each pair of strains (boxed experiments in part B) were averaged together and the average data for all 5 pairs were then averaged. Error bars represent standard deviations. (D) Individual replicates of competition experiments between phosphomutant (PM) and WT strains. Each box includes two-three replicates of one pair of strains (defined in part A). Average data for all 10 pairs of strains is presented in Fig 1B. (PDF) [file pgen.1010349.s001.pdf]

A

Competition	GFP- isolate	GFP- genotype	GFP+ isolate	GFP+ genotype
WT15	YMC449-2	*YHP1 YOX1*	YMC483-2	*YHP1 YOX1*
WT16	YMC449-1	*YHP1 YOX1*	YMC483-1	*YHP1 YOX1*
WT10	YMC449-4	*YHP1 YOX1*	YMC570-1	*YHP1 YOX1*
WT11	YMC522-1	*YHP1 YOX1*	YMC569-1	*YHP1 YOX1*
WT12	YMC522-2	*YHP1 YOX1*	YMC568-1	*YHP1 YOX1*
PM13	YMC473	*yhp1-13A yox1-9A*	YMC483-2	*YHP1 YOX1*
PM14	YMC449-1	*YHP1 YOX1*	YMC450-2	*yhp1-13A yox1-9A*
PM1	YMC473-1	*yhp1-13A yox1-9A*	YMC570-1	*YHP1 YOX1*
PM2	YMC572-1	*yhp1-13A yox1-9A*	YMC483-1	*YHP1 YOX1*
PM3	YMC572-2	*yhp1-13A yox1-9A*	YMC570-2	*YHP1 YOX1*
PM4	YMC572-3	*yhp1-13A yox1-9A*	YMC569-2	*YHP1 YOX1*
PM5	YMC573-1	*yhp1-13A yox1-9A*	YMC568-2	*YHP1 YOX1*
PM7	YMC574-2	*yhp1-13A yox1-9A*	YMC568-1	*YHP1 YOX1*
PM8	YMC574-3	*yhp1-13A yox1-9A*	YMC569-1	*YHP1 YOX1*
PM9	YMC574-4	*yhp1-13A yox1-9A*	YMC571-1	*YHP1 YOX1*

B

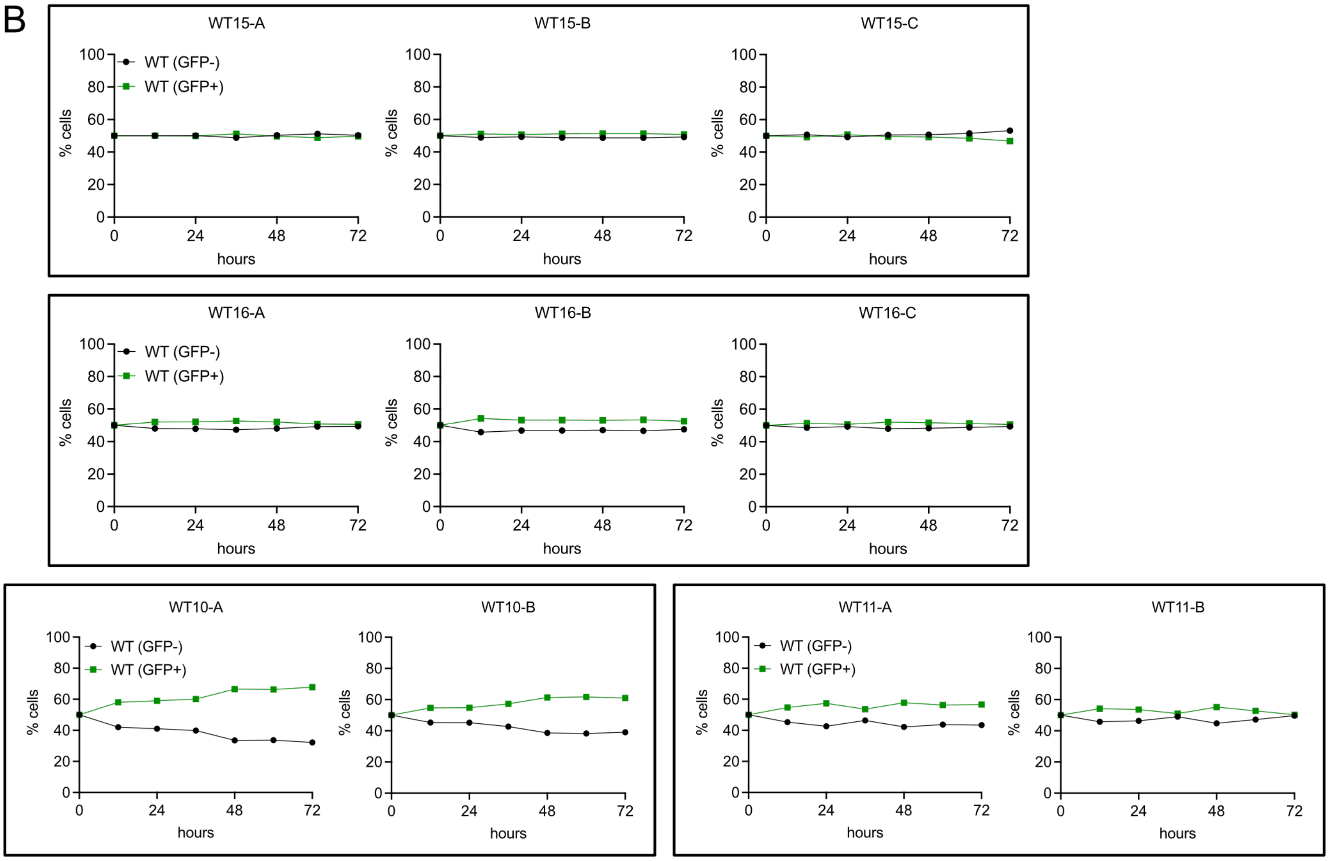

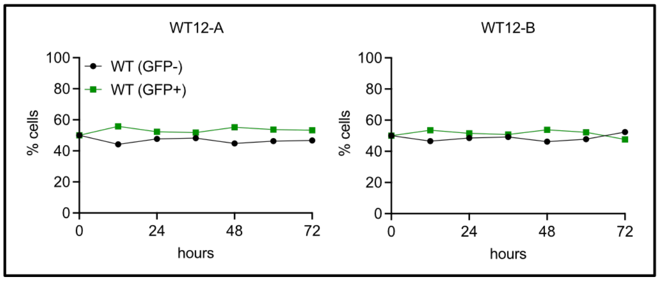

C

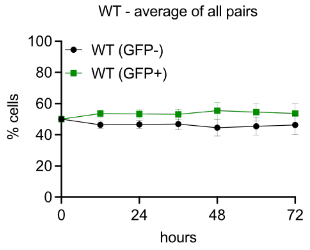

D

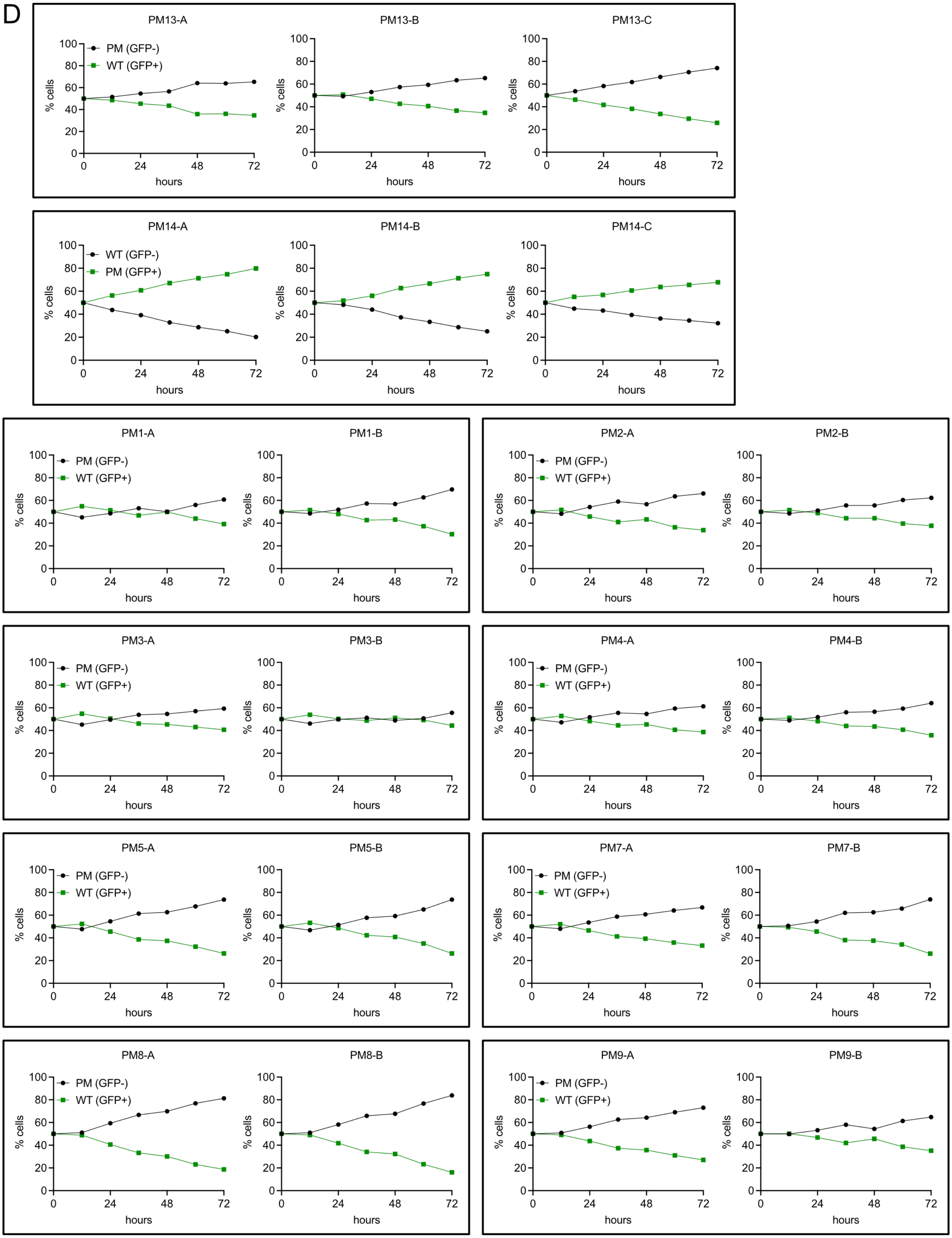
PM13-A
PM13-B
PM13-C
PM (GFP-)
WT (GFP+)
% cells
hours
PM14-A
PM14-B
PM14-C
WT (GFP-)
PM (GFP+)
PM1-A
PM1-B
PM2-A
PM2-B
PM3-A
PM3-B
PM4-A
PM4-B
PM5-A
PM5-B
PM7-A
PM7-B
PM8-A
PM8-B
PM9-A
PM9-B
