# Supplementary figures and images for: Repression of essential cell cycle genes increases cellular fitness
Source: PLoS Genet. 2022 Aug 29;18(8):e1010349. doi: 10.1371/journal.pgen.1010349 (PMC9462756; doi:10.1371/journal.pgen.1010349)

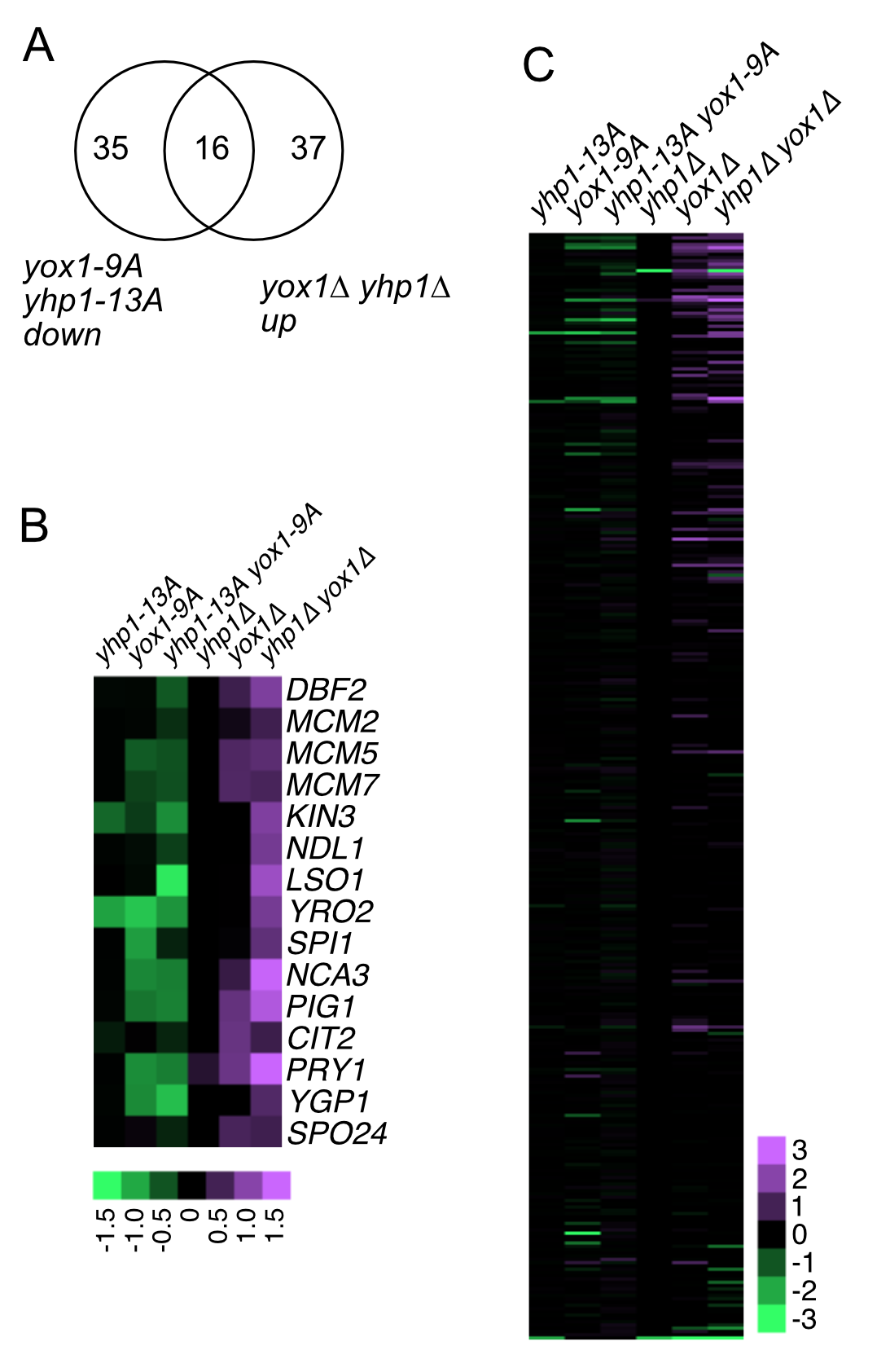

Supplement: S2 Fig — RNA-seq data for asynchronously growing yhp1 yox1 mutant strains. The indicated mutations are integrated at the endogenous locus of each gene. (A) Overlap of genes significantly downregulated in yhp1-13A yox1-9A and those significantly upregulated in yhp1Δ yox1Δ. Yhp1/Yox1 target genes should be in this category. List of genes is included in S5 Dataset. (B) Heat map showing log fold change of the 16 overlapping genes from part (A). (C) Heat map showing log2 fold change of all Yox1/Yhp1-regulated genes (defined as genes that were significantly changed upon acute overexpression of Yox1 or Yhp1, in Fig 2) in asynchronous cells with the indicated mutations integrated at each endogenous locus. Order of genes and scale matches Fig 2B. (TIF) [file pgen.1010349.s002.tif]

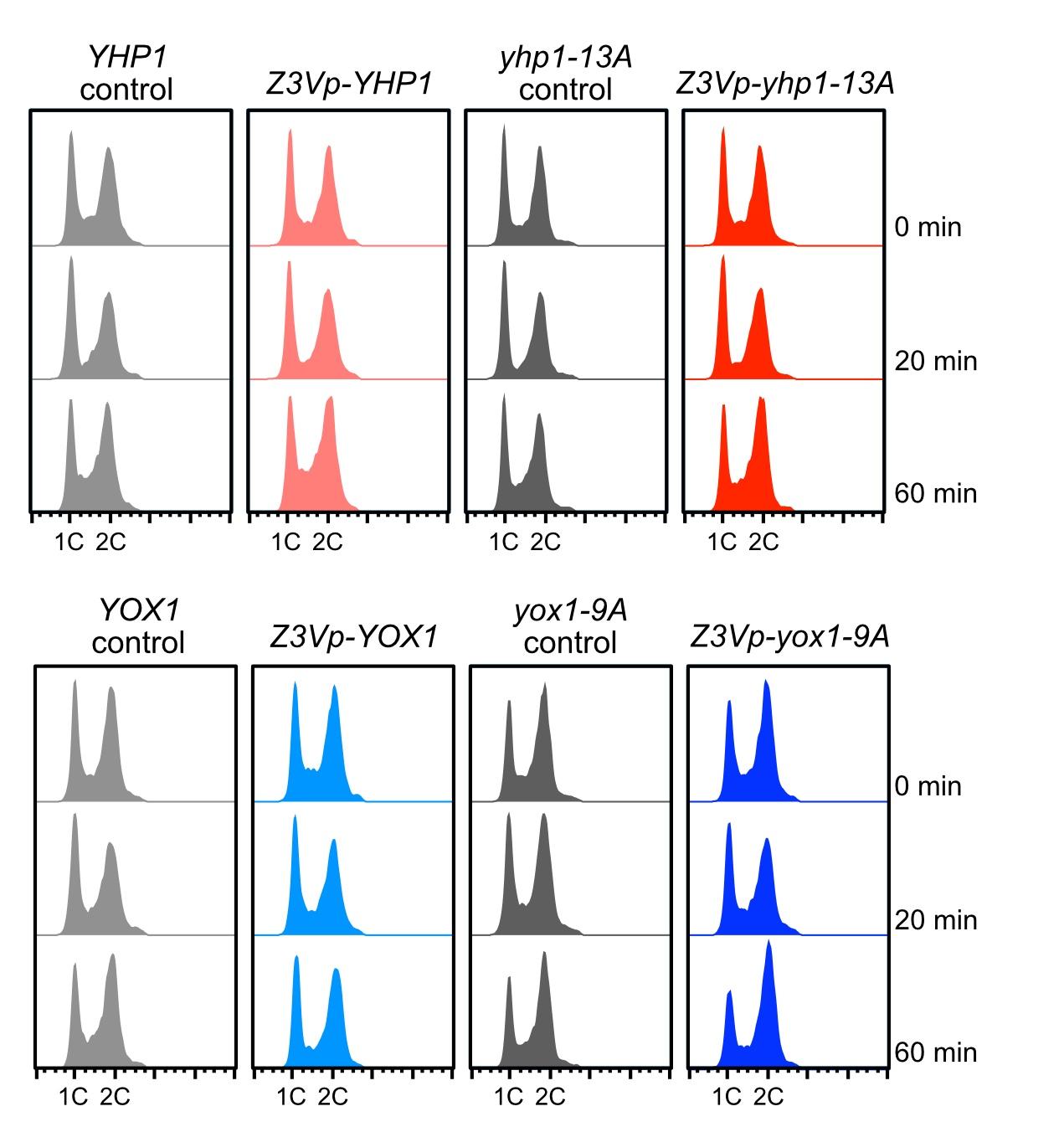

Supplement: S3 Fig — Representative FACS plots showing DNA content of strains following estradiol-induced expression of Yox1/Yhp1, phosphomutant proteins, or control strains. Control for Fig 2. (TIF) [file pgen.1010349.s003.tif]

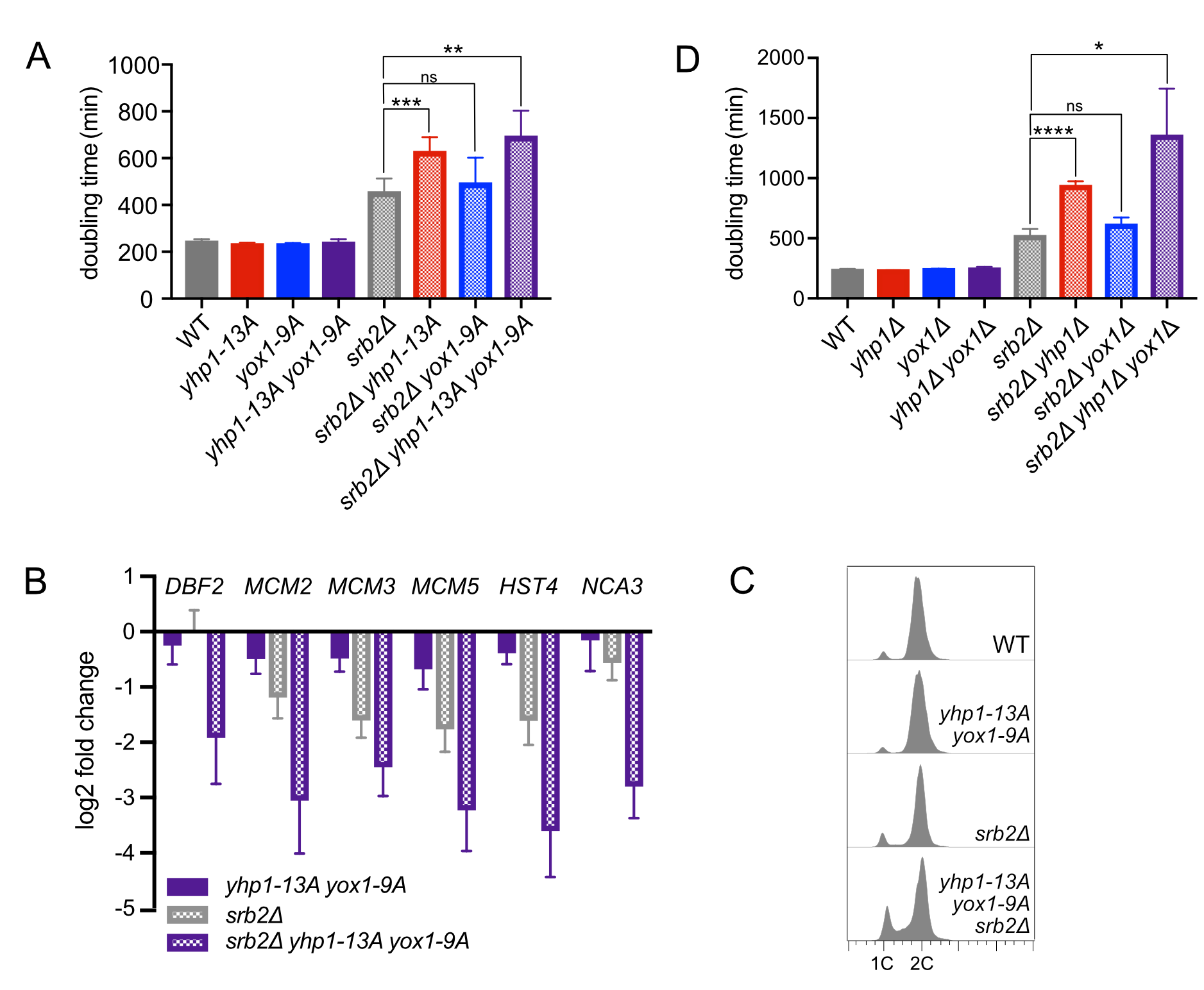

Supplement: S4 Fig — (A) Doubling times of the indicated strains growing at 30°C in synthetic medium. Shown is an average of n = 3–8 replicates, error bars indicate standard deviations. Significance was tested with an unpaired t-test, ** p < 0.005, ***p < 0.0005, ns = nonsignificant. (B) RT-qPCR of Yhp1/Yox1 target genes in the indicated strains that were synchronized in late S-phase. Shown are average log2 fold change values, compared to a wild type strain, in n = 3 experiments. Error bars indicate standard deviations. (C) Representative FACS plots of strains with the indicated genotypes from (B). (D) Doubling time of the indicated strains grown in synthetic medium at 30°C. Shown is an average of n = 3–4 replicates. Significance was tested using an unpaired t-test, *p < 0.05, ****p < 0.0001, ns = nonsignificant. (TIF) [file pgen.1010349.s004.tif]

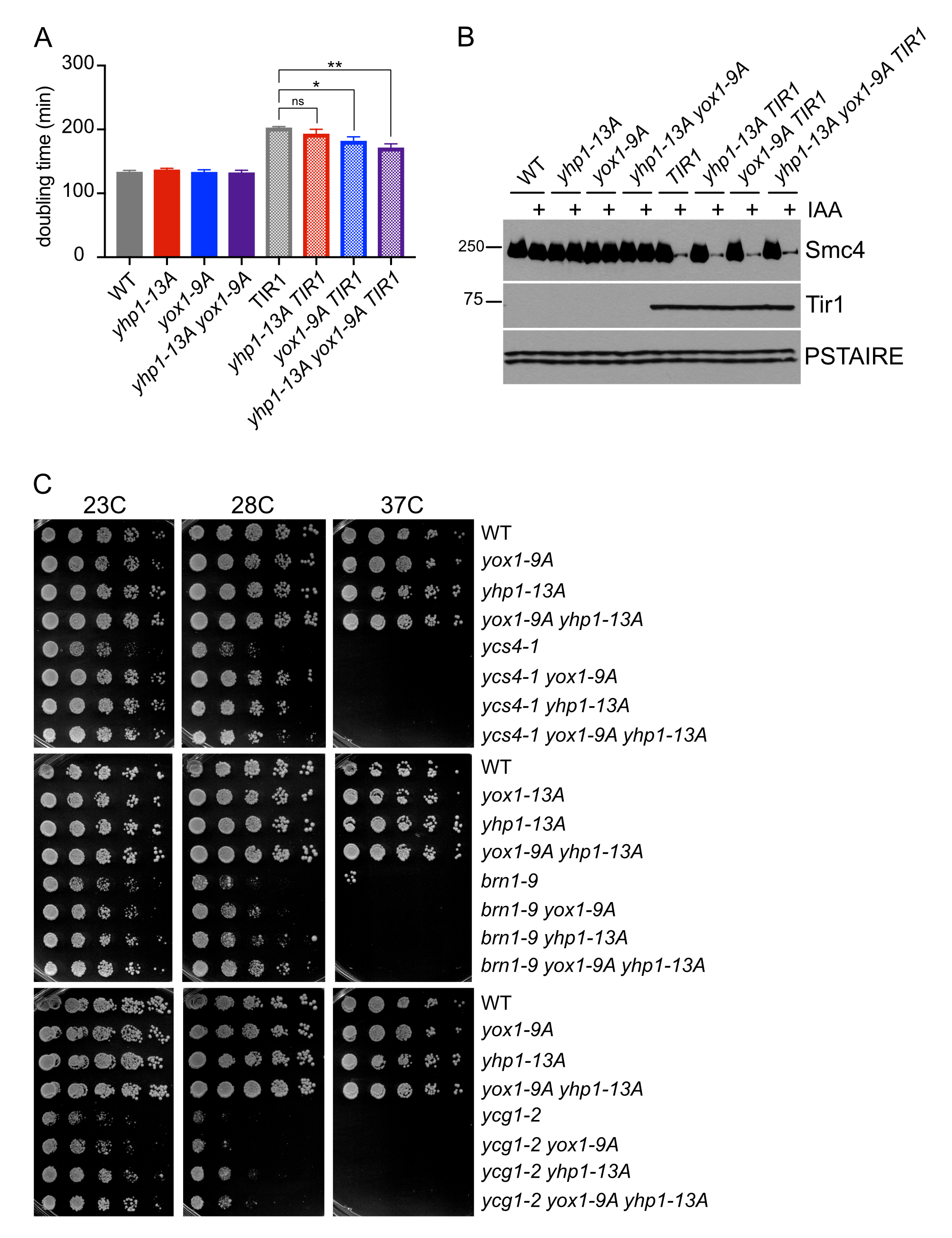

Supplement: S5 Fig — (A) Doubling times of Smc4-AID expressing strains with the indicated genotypes grown in rich medium containing 0.3 mM IAA (to degrade SMC4-AID). Shown is an average of n = 3 experiments. Significance was tested using an unpaired t-test, *p < 0.05, **p < 0.005, ns = nonsignificant. (B) Western blot of strains from (A) with or without the addition of 0.3mM IAA, as indicated. Smc4 is detected by a FLAG tag, TIR1 is detected by 3HA tag, PSTAIRE is shown as a loading control. (C) Five-fold dilutions of strains with the indicated genotypes were plated on rich medium and grown at the indicated temperatures. (TIF) [file pgen.1010349.s005.tif]

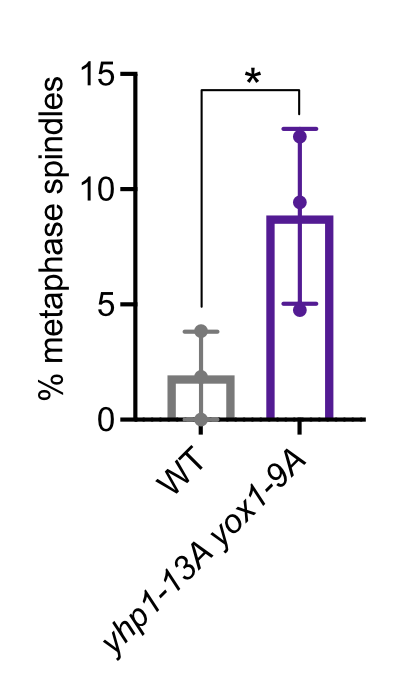

Supplement: S6 Fig — Quantitation of metaphase spindles at the 60-minute time point following G1 arrest-release, as performed in Fig 5A. Shown in the mean percentage of metaphase spindles from n = 3 experiments. Significance was tested using a paired t-test, *p = 0.0247. (TIFF) [file pgen.1010349.s006.tiff]

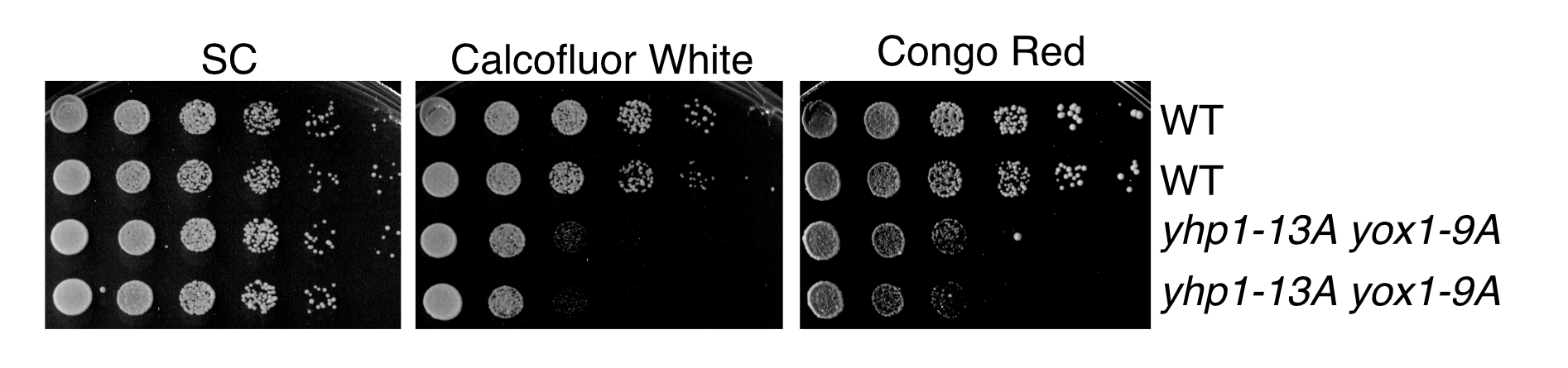

Supplement: S7 Fig — Five-fold dilutions of wild type or yhp1-13A yox1-9A cells were plated on synthetic complete (SC) medium, or SC medium containing 150 μg/mL Calcofluor White or 0.25 mg/mL Congo Red to elicit cell wall stress. Two isolates of each genotype are shown. (TIF) [file pgen.1010349.s007.tif]

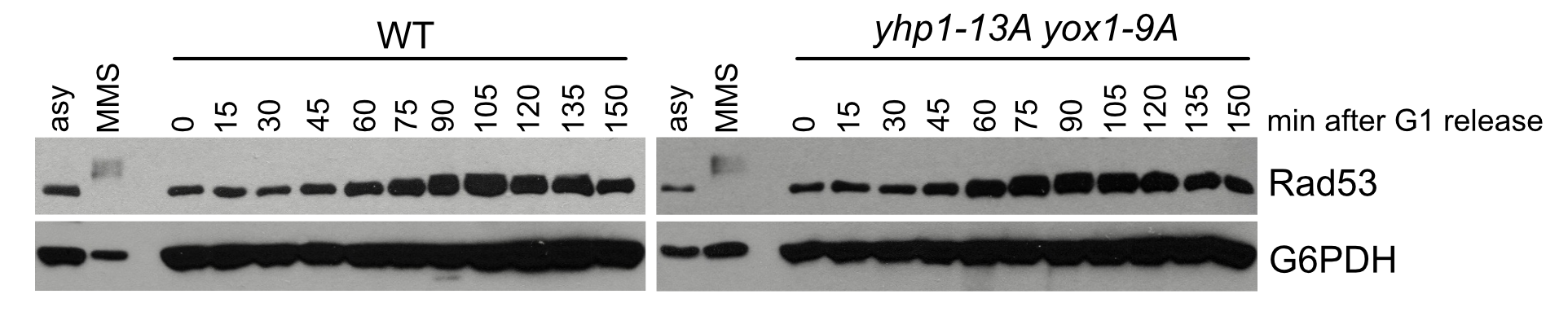

Supplement: S8 Fig — Cells with the indicated genotypes were arrested in G1 with alpha-factor and released into the cell cycle as in Fig 5A. Western blot of Rad53 is shown to determine if phosphorylation can be detected, which would be an indication of DNA damage. Lysates from untreated asynchronous (asy) and MMS-treated (0.05% for 3 hours) wild type cells are included as an example of a DNA-damage induced Rad53 phosphoshift. G6PDH is shown as a loading control. (TIF) [file pgen.1010349.s008.tif]
