# Supplementary material for: Repression of essential cell cycle genes increases cellular fitness
Source: PLoS Genet. 2022 Aug 29;18(8):e1010349. doi: 10.1371/journal.pgen.1010349 (PMC9462756; doi:10.1371/journal.pgen.1010349)
Supplement: S1 Table — Shown are significant genetic interaction values with a p-value of < 0.05. Strong interactionswith a |ε| ≥ 0.08 are plotted in Fig 6D. (PDF) [file pgen.1010349.s009.pdf]

S1 Table. Genetic Interactions between condensin alleles and mitotic exit regulators

	brn1-16	brn1-9	smc2-8	smc4-1	ycg1-2	ycs4-1	category
**apc1-5004**		-0.1285		-0.1591			APC(Cdc20)
**apc11-13**			0.0947	0.0641	0.0834		APC(Cdc20)
**apc2-8**		0.0639	0.0733				APC(Cdc20)
**apc4-5001**							APC(Cdc20)
**apc5-ca**		0.1058	0.0822		0.1564	0.1464	APC(Cdc20)
**apc5-ca-paps**	0.0836	0.0686	0.1173	0.1091	0.1468	0.1371	APC(Cdc20)
**apc9**		0.0855					APC(Cdc20)
**cdc16-1**		0.1097	0.0413	0.1033	0.1095	0.0728	APC(Cdc20)
**cdc20-1**		0.1144	0.1166	0.2459	0.2236		APC(Cdc20)
**cdc20-2**	0.1472		0.0456	0.1923			APC(Cdc20)
**cdc20-3**	0.1287			0.1866	0.2067		APC(Cdc20)
**cdc23-1**	0.0505			0.3127			APC(Cdc20)
**cdc23-4**		0.0816			0.1855		APC(Cdc20)
**cdc27-1**			0.0704		0.1657	0.1101	APC(Cdc20)
**cdc27-2**			0.0681	0.087	0.0929	0.1117	APC(Cdc20)
**mnd2**							APC(Cdc20)
**swm1**		0.0691		0.0845		0.1182	APC(Cdc20)
**bns1**	0.0311						FEAR
**cdc55-supp1**				-0.1615	0.0412		FEAR
**esp1-1**	-0.2959	-0.2639		-0.1037	-0.5802	-0.3402	FEAR
**fob1**	-0.2896	-0.3902			-0.2574	-0.5002	FEAR
**pds1-128**			-0.0932				FEAR
**slk19**		-0.1772				-0.1833	FEAR
**spo12**							FEAR
**zds1**		0.0309					FEAR
**bfa1**					-0.1499		MEN
**bub2**							MEN
**cdc15-1**		0.1175	0.0487	0.1734	0.122	0.1069	MEN
**cdc15-2**			-0.1182			0.1672	MEN
**dbf2**		0.055		0.1485		0.1096	MEN
**dbf2-1**		0.0986	0.0459	0.0819	0.1006	0.117	MEN
**dbf2-2**	0.0592	0.1145	0.0522	0.0698	0.1656	0.0665	MEN
**dbf2-3**		0.1413		0.0971		0.16	MEN
**dbf20**							MEN
**lte1**							MEN
**lte1-supp1**	0.074	0.1376		0.0871		0.1392	MEN
**mob1-5001**	0.0478	0.0745		0.1138	0.1579	0.0713	MEN
**tem1-3**		0.0967		0.1957			MEN
**tem1-3-supp1**		0.0305					MEN
**cdc14-1**		-0.2795		-0.1851	-0.2588	-0.288	CDC14
**cdc14-2**		-0.3251		-0.216		-0.3192	CDC14
**cdc14-8**		-0.1575		0.0425		-0.2831	CDC14
