# Supplementary material for: Repression of essential cell cycle genes increases cellular fitness
Source: PLoS Genet. 2022 Aug 29;18(8):e1010349. doi: 10.1371/journal.pgen.1010349 (PMC9462756; doi:10.1371/journal.pgen.1010349)
Supplement: S2 Table — All S. cerevisiae strains are in the BY4741 background. (PDF) [file pgen.1010349.s010.pdf]

S2 Table. Strain table

strain	genotype	figure
YMC72	*MATa his3Δ1 ura3Δ0 leu2Δ0 met15Δ0*	1A, 3D, S4A,D, S7
YMC68	*MATa his3Δ1 ura3Δ0 leu2Δ0 met15Δ0 yhp1-13A-13MYC-LEU2*	1A, 3D, S4A
YMC70	*MATa his3Δ1 ura3Δ0 leu2Δ0 met15Δ0 yox1-9A-3V5-NatMX*	1A, 3D, S4A
YMC67	*MATa his3Δ1 ura3Δ0 leu2Δ0 met15Δ0 yhp1-13A-13MYC-LEU2 yox1-9A-3V5-NatMX*	1A, 3D, S4A-C, S7
YMC449 YMC522	*MATa his3Δ1 ura3Δ0 leu2Δ0 met15Δ0 YHP1-13MYC-LEU2 YOX1-3V5-NatMX chrVIΔ181901-182001::Hyg-TEFp-GFP(Y66F)*	1B, S1
YMC450	*MATa his3Δ1 ura3Δ0 leu2Δ0 met15Δ0 yhp1-13A-13MYC-LEU2 yox1-9A-3V5-NatMX chrVIΔ181901-182001::Hyg-TEFp-GFP*	1B, S1
YMC473 YMC572 YMC573 YMC574	*MATa his3Δ1 ura3Δ0 leu2Δ0 met15Δ0 yhp1-13A-13MYC-LEU2 yox1-9A-3V5-NatMX chrVI181901-182001Δ::Hyg-TEFp-GFP(Y66F)*	1B, S1
YMC483 YMC568 YMC569 YMC570 YMC571	*MATa his3Δ1 ura3Δ0 leu2Δ0 met15Δ0 YHP1-13MYC-LEU2 YOX1-3V5-NatMX chrVI181901-182001Δ::Hyg-TEFp-GFP*	1B, S1
YCL64	*MATα his3Δ1 ura3Δ0 leu2Δ0 met15Δ0 yhp1-13A-13MYC-LEU2 yox1-9A-3V5-NatMX can1Δ::STE2p-Sphis5 lyp1Δ*	1D, 1F, 1H
YMC124	*MATα his3Δ1 ura3Δ0 leu2Δ0 met15Δ0 yhp1Δ::LEU2 yox1Δ::NatMX can1Δ::STE2p-Sphis5 lyp1Δ*	1D, 1G-H
YAG180	*MATa URA3::Z4p-YOX1-3V5-KanMX HAP1::NatMX::ACT1p-Z3EV-ENO2term ura3Δ0 can1ΔSte2p-Sphis5 his3Δ1 lypΔ0*	2, S3
YAG181	*MATa URA3::Z4p-HO HAP1::NatMX::ACT1p-Z3EV-ENO2term YOX1-3V5-KanMX ura3Δ0 can1ΔSte2p-Sphis5 his3Δ1 lypΔ0*	2, S3
YMC213	*MATa URA3::Z4p-yox1-9A-3V5-KanMX HAP1::NatMX::ACT1p-Z3EV-ENO2term ura3Δ0 can1ΔSte2p-Sphis5 his3Δ1 lypΔ0*	2, S3
YMC214	*MATa URA3::Z4p-HO HAP1::NatMX::ACT1p-Z3EV-ENO2term yox1-9A-3V5-KanMX ura3Δ0 can1ΔSte2p-Sphis5 his3Δ1 lypΔ0*	2, S3
YMC228	*MATa URA3::Z4p-HO HAP1::NatMX::ACT1p-Z3EV-ENO2term YHP1-3V5-KanMX ura3Δ0 can1ΔSte2p-Sphis5 his3Δ1 lypΔ0*	2, S3
YMC229	*MATa URA3::Z4p-HO HAP1::NatMX::ACT1p-Z3EV-ENO2term yhp1-13A-3V5-KanMX ura3Δ0 can1ΔSte2p-Sphis5 his3Δ1 lypΔ0*	2, S3
YMC230	*MATa URA3::Z4p-YHP1-3V5-KanMX HAP1::NatMX::ACT1p-Z3EV-ENO2term ura3Δ0 can1ΔSte2p-Sphis5 his3Δ1 lypΔ0*	2, S3
YMC231	*MATa URA3::Z4p-yhp1-13A-3V5-KanMX HAP1::NatMX::ACT1p-Z3EV-ENO2term ura3Δ0 can1ΔSte2p-Sphis5 his3Δ1 lypΔ0*	2, S3
YMC451	*MATa his3Δ1 ura3Δ0 leu2Δ0 met15Δ0 dbf2-2::KanMX*	3D
YMC452	*MATa his3Δ1 ura3Δ0 leu2Δ0 met15Δ0 dbf2-2::KanMX yhp1-13A-13MYC-LEU2*	3D
YMC137	*MATa his3Δ1 ura3Δ0 leu2Δ0 met15Δ0 dbf2-2::KanMX yox1-9A-3V5-NatMX*	3D

YMC453	*MATa his3Δ1 ura3Δ0 leu2Δ0 met15Δ0 dbf2-2::KanMX yhp1-13A-13MYC-LEU2 yox1-9A-3V5-NatMX*	3D
YMC59	*MATa his3Δ1 ura3Δ0 leu2Δ0 met15Δ0 srb2Δ::KanMX*	S4
YMC63	*MATa his3Δ1 ura3Δ0 leu2Δ0 met15Δ0 srb2Δ::KanMX yhp1-13A-13MYC-LEU2*	S4A
YMC61	*MATa his3Δ1 ura3Δ0 leu2Δ0 met15Δ0 srb2Δ::KanMX yox1-9A-3V5-NatMX*	S4A
YMC65	*MATa his3Δ1 ura3Δ0 leu2Δ0 met15Δ0 srb2Δ::KanMX yhp1-13A-13MYC-LEU2 yox1-9A-3V5-NatMX*	S4A-C
YBL307	*MATa his3Δ1 ura3Δ0 leu2Δ0 met15Δ0 YHP1-13MYC-LEU2 YOX1-3V5-KanMX*	4B, S2, S4, S5C
YFS105	h- leu1-32 ura4D18 (*S.pombe*)	S4B
YBL246	*MATa his3Δ1 ura3Δ0 leu2Δ0 met15Δ0 yox1-9A-3V5-KanMX*	4B, S2, S5C
YBL242	*MATa his3Δ1 ura3Δ0 leu2Δ0 met15Δ0 yhp1-13A-13MYC-LEU2*	4B, S2, S5C
YBL261	*MATa his3Δ1 ura3Δ0 leu2Δ0 met15Δ0 yhp1-13A-13MYC-LEU2 yox1-9A-3V5-KanMX*	4B, S2, S5C
Y9304	*MATa his3Δ1 ura3Δ0 leu2Δ0 met15Δ0 ycs4-1::KanMX*	4B, S5C, 6E
YAG53	*MATa his3Δ1 ura3Δ0 leu2Δ0 met15Δ0 ycs4-1::KanMX yox1-9A-3V5-NatMX*	4B, S5C
YAG52	*MATa his3Δ1 ura3Δ0 leu2Δ0 met15Δ0 ycs4-1::KanMX yhp1-13A-13MYC-LEU2*	4B, S5C
YAG43	*MATa his3Δ1 ura3Δ0 leu2Δ0 met15Δ0 ycs4-1::KanMX yhp1-13A-13MYC-LEU2 yox1-9A-3V5-NatMX*	4B, S5C
Y9804	*MATa his3Δ1 ura3Δ0 leu2Δ0 met15Δ0 brn1-9::KanMX*	4B, S5C
YAG55	*MATa his3Δ1 ura3Δ0 leu2Δ0 met15Δ0 brn1-9::KanMX yox1-9A-3V5-NatMX*	4B, S5C
YAG66	*MATa his3Δ1 ura3Δ0 leu2Δ0 met15Δ0 brn1-9::KanMX yhp1-13A-13MYC-LEU2*	4B, S5C
YAG45	*MATa his3Δ1 ura3Δ0 leu2Δ0 met15Δ0 brn1-9::KanMX yhp1-13A-13MYC-LEU2 yox1-9A-3V5-NatMX*	4B, S5C
Y10100	*MATa his3Δ1 ura3Δ0 leu2Δ0 met15Δ0 ycg1-2::KanMX*	4B, S5C
YAG59	*MATa his3Δ1 ura3Δ0 leu2Δ0 met15Δ0 ycg1-2::KanMX yox1-9A-3V5-NatMX*	4B, S5C
YAG60	*MATa his3Δ1 ura3Δ0 leu2Δ0 met15Δ0 ycg1-2::KanMX yhp1-13A-13MYC-LEU2*	4B, S5C
YAG49	*MATa his3Δ1 ura3Δ0 leu2Δ0 met15Δ0 ycg1-2::KanMX yhp1-13A-13MYC-LEU2 yox1-9A-3V5-NatMX*	4B, 4E, S5C
YJG77	*MATa his3Δ1 ura3Δ0 leu2Δ0 met15Δ0*	4C,D, 6E
YJG126	*MATa his3Δ1 ura3Δ0 leu2Δ0 met15Δ0 yhp1Δ::LEU2 yox1Δ::NatMX*	4C,D
YJG81	*MATa his3Δ1 ura3Δ0 leu2Δ0 met15Δ0 ycg1-2::KanMX*	4C,E, 6E
YJG82	*MATα his3Δ1 ura3Δ0 leu2Δ0 lys2Δ0 ycg1-2::KanMX*	4C, 6E
YJG112	*MATa his3Δ1 ura3Δ0 leu2Δ0 met15Δ0 yhp1Δ::LEU2 yox1Δ::NatMX ycg1-2::KanMX*	4C
YJG87	*MATa his3Δ1 ura3Δ0 leu2Δ0 met15Δ0 ycs4-1::KanMX*	4D, 6E
YJG88	*MATα his3Δ1 ura3Δ0 leu2Δ0 lys2Δ0 ycs4-1::KanMX*	4D, 6E

YJG120	*MATa his3Δ1 ura3Δ0 leu2Δ0 met15Δ0 yhp1Δ::LEU2 yox1Δ::NatMX ycs4-1::KanMX*	4D
YMC101	*MATa his3Δ1 ura3Δ0 leu2Δ0 met15Δ0 YHP1-13MYC-LEU2 YOX1-3V5-NatMX*	4E
YJG94	*MATa his3Δ1 ura3Δ0 leu2Δ0 met15Δ0 yhp1-13A-13MYC-LEU2 yox1-9A-3V5-NatMX*	4E
YJG52	*MATa his3Δ1 ura3Δ0 leu2Δ0 met15Δ0 yhp1-13A-13MYC-LEU2 yox1-9A-3V5-NatMX 3HA-CDC14*	5A-C, S6
YJG102	*MATa his3Δ1 ura3Δ0 leu2Δ0 met15Δ0 YHP1-13MYC-LEU2 YOX1-3V5-NatMX 3HA-CDC14*	5A-C, S6
YJG110	*MATa his3Δ1 ura3Δ0 leu2Δ0 met15Δ0 yhp1-13A-13MYC-LEU2 yox1-9A-3V5-NatMX 3HA-CDC14 FAR1-3V5- KanMX*	5D, S8
YJG111	*MATa his3Δ1 ura3Δ0 leu2Δ0 met15Δ0 YHP1-13MYC-LEU2 YOX1-3V5-NatMX 3HA-CDC14 FAR1-3V5-KanMX*	5D, S8
YMC132	*MATa his3Δ1 ura3Δ0 leu2Δ0 met15Δ0 yhp1Δ::LEU2*	S4D
YJG84	*MATα his3Δ1 ura3Δ0 leu2Δ0 lys2Δ0 dbf2Δ::URA3*	6E
YJG89	*MATa his3Δ1 ura3Δ0 leu2Δ0 lys2Δ0 dbf2Δ::URA3*	6E
YJG79	*MATa his3Δ1 ura3Δ0 leu2Δ0 met15Δ0 ycg1-2::KanMX dbf2Δ::URA3*	6E
YJG80	*MATα his3Δ1 ura3Δ0 leu2Δ0 lys2Δ0 ycg1-2::KanMX dbf2Δ::URA3*	6E
YJG85	*MATa his3Δ1 ura3Δ0 leu2Δ0 met15Δ0 ycs4-1::KanMX dbf2Δ::URA3*	6E
YJG86	*MATα his3Δ1 ura3Δ0 leu2Δ0 met15Δ0 lys2Δ0 ycs4-1::KanMX dbf2Δ::URA3*	6E
YBL330	*MATa his3Δ1 ura3Δ0 leu2Δ0 lys2Δ0 yhp1Δ::LEU2*	S2
YBL334	*MATa his3Δ1 ura3Δ0 leu2Δ0 met15Δ0 yox1Δ::KanMX*	S2
YMC134	*MATa his3Δ1 ura3Δ0 leu2Δ0 met15Δ0 yox1Δ::NatMX*	S4D
YMC125	*MATa his3Δ1 ura3Δ0 leu2Δ0 met15Δ0 yhp1Δ::LEU2 yox1Δ::NatMX*	S2, S4D
YMC363	*MATa his3Δ1 ura3Δ0 leu2Δ0 met15Δ0 yhp1Δ::LEU2 srb2Δ::KanMX*	S4D
YMC365	*MATa his3Δ1 ura3Δ0 leu2Δ0 met15Δ0 yox1Δ::NatMX srb2Δ::KanMX*	S4D
YMC361	*MATa his3Δ1 ura3Δ0 leu2Δ0 met15Δ0 yhp1Δ::LEU2 yox1Δ::NatMX srb2Δ::KanMX*	S4D
YMC268	*MATa his3Δ1 ura3Δ0 leu2Δ0 met15Δ0 SMC4-AID*-6FLAG-Hyg*	S5A, S5B
YMC264	*MATa his3Δ1 ura3Δ0 leu2Δ0 met15Δ0 SMC 4-AID*-6FLAG-Hyg yhp1-13A-13MYC-LEU2*	S5A, S5B
YMC266	*MATa his3Δ1 ura3Δ0 leu2Δ0 met15Δ0 SMC 4-AID*-6FLAG-Hyg yox1-9A-3V5-NatMX*	S5A, S5B
YMC262	*MATa his3Δ1 ura3Δ0 leu2Δ0 met15Δ0 SMC 4-AID*-6FLAG-Hyg yhp1-13A-13MYC-LEU2 yox1-9A-3V5-NatMX*	S5A, S5B
YMC258	*MATa his3Δ1 ura3Δ0 leu2Δ0 met15Δ0 SMC 4-AID*-6FLAG-Hyg chrVIΔ181901-182001::TIR1-3HA-KanMX*	S5A, S5B
YMC254	*MATa his3Δ1 ura3Δ0 leu2Δ0 met15Δ0 SMC 4-AID*-6FLAG-Hyg chrVIΔ181901-182001::TIR1-3HA-KanMX yhp1-13A-13MYC-LEU2*	S5A, S5B
YMC256	*MATa his3Δ1 ura3Δ0 leu2Δ0 met15Δ0 SMC 4-AID*-6FLAG-Hyg chrVIΔ181901-182001::TIR1-3HA-KanMX yox1-9A-3V5-NatMX*	S5A, S5B

YMC252	*MATa his3Δ1 ura3Δ0 leu2Δ0 met15Δ0 smc4-AID*-6FLAG-Hyg chrVIΔ181901-182001::TIR1-3HA-KanMX yhp1-13A-13MYC-LEU2 yox1-9A-3V5-NatMX*	S5A, S5B
YJG58	*MATa his3Δ1 ura3Δ0 leu2Δ0 met15Δ0 yhp1-13A-13Myc-LEU2 yox1-9A-3V5-NatMX*	5E
YJG108	*MATa his3Δ1 ura3Δ0 leu2Δ0 met15Δ0 YHP1-13MYC-LEU2 YOX1-3V5-NatMX*	5E
YJG185	*MATa his3Δ1 ura3Δ0 leu2Δ0 met15Δ0 yhp1-13A-13MYC-LEU2 yox1-9A-3V5-NatMX far1Δ::KanMX*	5E
YJG186	*MATa his3Δ1 ura3Δ0 leu2Δ0 met15Δ0 YHP1-13MYC-LEU2 YOX1-3V5-NatMX far1Δ::KanMX*	5E

All *S. cerevisiae* strains are in the BY4741 background.
