# Supplementary material for: Repression of essential cell cycle genes increases cellular fitness
Source: PLoS Genet. 2022 Aug 29;18(8):e1010349. doi: 10.1371/journal.pgen.1010349 (PMC9462756; doi:10.1371/journal.pgen.1010349)
Supplement: S3 Table — List of all primers used in RT-qPCR experiments. (PDF) [file pgen.1010349.s011.pdf]

S3 Table. Primer table

gene	species	primer name	sequence
ACT1	*S. pombe*	ACT1sp FWD1	ATCCAACCGTGAGAAGATGACT
ACT1	*S. pombe*	ACT1sp REV1	AAATGGGAACAGTGTGGGTAAC
DBF2	*S. cerevisiae*	Dbf2-Fwd	GTCTCCGGATTATATGGCTTTG
DBF2	*S. cerevisiae*	Dbf2-Rev	GCGCCTTAAGTTGTCATAGGTC
MCM2	*S. cerevisiae*	MCM2fwd	GGCTAACAGTTACTCGGAATGG
MCM2	*S. cerevisiae*	MCM2rev	AGACACTTAGCGGAGTCCAAAG
MCM3	*S. cerevisiae*	MCM3fwd	ACATTGATCGGGTTCAAAACTC
MCM3	*S. cerevisiae*	MCM3rev	AGGCGCTAAAGATTGAGACAAG
MCM5	*S. cerevisiae*	MCM5 FWD	ACCGTCAGTTTACCACGTTCTT
MCM5	*S. cerevisiae*	MCM5 REV	TACAGGAAATCCCAGAACTGGT
HST4	*S. cerevisiae*	Hst4-Fwd	GATTATCCAAGAATTGCCAACC
HST4	*S. cerevisiae*	Hst4-Rev	GTACTGTACTGGGTATTGGCTTC
NCA3	*S. cerevisiae*	NCA3 FWD	ATGTTCGGACTTCCCTTCTGTA
NCA3	*S. cerevisiae*	NCA3 REV	AATGTCAAAGACTCAATGGCCT
